# Negativity Spreads More than Positivity on Twitter After Both Positive and Negative Political Situations

**DOI:** 10.1007/s42761-021-00057-7

**Published:** 2021-10-12

**Authors:** Jonas Paul Schöne, Brian Parkinson, Amit Goldenberg

**Affiliations:** 1 Department of Experimental Psychology, University of Oxford, Oxford, England; 2 Harvard Business School, Harvard University, Oxford, England

**Keywords:** Emotion contagion, Social media, Emotions, Politics, Intergroup

## Abstract

What type of emotional language spreads further in political discourses on social media? Previous research has focused on situations that primarily elicited negative emotions, showing that negative language tended to spread further. The current project extends existing knowledge by examining the spread of emotional language in response to both predominantly positive and negative political situations. In Study 1, we examined the spread of emotional language in tweets related to the winning and losing parties in the 2016 US elections, finding that increased negativity (but not positivity) predicted content sharing in both situations. In Study 2, we compared the spread of emotional language in two separate situations: the celebration of the US Supreme Court approval of same-sex marriage (positive) and the Ferguson unrest (negative), finding again that negativity spread further. These results shed light on the nature of political discourse and engagement.

**Supplementary Information:**

The online version contains supplementary material available at 10.1007/s42761-021-00057-7.

On June 26, 2015, the US Supreme Court reached a 5:4 decision that all states in the USA were required to grant same-sex marriages. This decision was the culmination of a collective action movement directed at allowing gay couples an equal standing in front of the law. The decision was celebrated around the USA and the world. But supporters of the movement did not only use positive language to express happiness. Many also shared the frustration they had felt over the years or expressed anger towards those who opposed them. On Twitter, millions of both positive and negative tweets spread online under hashtags such as #loveWins and #SameLove.

The predominantly positive celebration in response to the same-sex marriage movement and other political celebrations provide an opportunity to learn about the spread of emotional language in response to more positive situations. Previous research on the spread of emotional language in political contexts has shown that negative language is more likely to spread than positive language (Brady et al., 2017; Goldenberg & Gross, 2020). But this research has primarily focused on political situations that elicited negative emotions such as school shootings (Doré et al., 2015) or anti-government movements (Alvarez et al., 2015). Learning about how emotional language spreads in response to political celebrations can complement these findings and provide a more complete view on the nature of political discourse online.

One prediction regarding the spread of emotional language in political celebrations is that positive language will spread further. This can be supported by some evidence that after a positive event, people are seeking others to share their positive emotions (Gable et al., 2004; Isen, 1984). However, these empirical findings did not focus on intergroup situations in which the celebrations were often associated with the loss of the other side. In these competitive intergroup situations, negative emotions towards the outgroup are likely to be prevalent and in some cases even exceed positive affect towards one’s group (Cikara, 2015; Finkel et al., 2020). Furthermore, group members often use negativity to signal other group members of one’s values and beliefs (Crockett, 2017; Jordan et al., 2016). The goals of the current research are to replicate previous findings derived from studies of negative political events and to empirically assess conflicting predictions about the spread of emotional language in positive political contexts.

## The Spread of Emotional Language in Response to Political Situations on Social Media

In an era in which much political discourse takes place mostly online (Aral, 2020; Tufekci, 2017), learning more about what type of language spreads further takes us a step closer to understanding what content lures people into participating in political discourse. But insights from such analyses are not limited to dynamics on social media, as the psychological constructs that guide online sharing are likely to be similar to those driving offline sharing. Social media provides opportunities to learn about these processes as they naturally unfold on a large scale.

The spread of certain kinds of linguistic content on social media can be assessed in multiple ways (for an overview, see Goldenberg & Gross, 2020). A common method is counting how many times a specific content is shared by other users. Unlike other forms of engagement such as liking or replying, sharing content without adding any text often indicates agreement with the conveyed message (Goldenberg & Gross, 2020). Another advantage of counting shares (or retweets) over alternative measures of spread is that it leaves little doubt that the user viewed the original post.

It is well established that emotional information is shared more frequently than non-emotional information, both offline and online (Rimé, 2007; Rimé et al., 1998; Stieglitz & Dang-Xuan, 2013). However, focus on political situations has mostly been on situations that elicited negative language (Brady et al., 2017; Doré et al., 2015; Garcia & Rimé, 2019). In these contexts, it has been found that negative language leads to more shares than positive language. However, political situations are not only negative and typically involve both winners and losers. Understanding the spread of emotional language in response to political victories can shed light on whether political discourse on even positive situations may contribute to intergroup hostility and polarization.

It may seem plausible that because positive language dominates political celebrations, it should also be more likely to be shared. Congruent with this prediction, Gable et al. (2004) found that people seek to share their positive feelings with others in response to positive events. This is done in order to increase positive social feedback from other people (Langston, 1994). However, available empirical support for this prediction is limited. One relevant study by Brady et al. (2017) investigated the spread of emotional language in tweets relating to the supreme court ruling for same-sex marriage in 2015. However, their analysis focused on tweets that were written 5 months after the ruling was made. Results from their analysis suggested that content was shared more often if it contained words referring to positive moral emotions. However, the time that has passed since the ruling may have led to the fact that the specific search terms used to generate the dataset (gay rights, gay wedding or love wins) may have already become part of a more general discourse concerning gay relationship and marriage. It is therefore crucial to examine the spread of emotional language soon after the target situation occurred.

By contrast, some accounts provide grounds for predicting that negative content should be shared more even in positive political contexts. First, increased attention to negative tweets may motivate users to further share these tweets. Previous work has suggested that increased attention to political posts is associated with further engagement (Brady et al., 2020). As negative emotions are more attention-grabbing than positive emotions (Meffert et al., 2006; Rozin & Royzman, 2001), it is possible that users attention to such content would render further engagement. Negative emotions may be especially attention-grabbing in intergroup contexts in which negative emotions towards the outgroup are often prevalent and may even exceed the intensity of positive emotions (Cikara, 2015). This is particularly true in the US political context, in which outgroup hate exceeds ingroup love and is central to group identification (Finkel et al., 2020). Twitter users may share outgroup-directed negative content more because they wish to signal other group members of their group membership and because they believe that ingroup members will relish reading it (Crockett, 2017; Jordan et al., 2016). The use of negative in intergroup context may be hedonically pleasing due to the support that users may get from other group members (Cohen-Chen et al., 2020).

Assuming that negative emotional language may indeed spread further even in positive political situations, one potential concern is that this is driven by intergroup rather than intragroup interactions in which negative content in response to political celebrations is produced by outgroup members. Assessing users’ political affiliation is required to evaluate the extent of intragroup versus intergroup interactions.

### The Present Research

The goal of the present research was to examine the spread of emotional language on Twitter in response to both positive and negative political situations. In Study 1, we tested the spread of emotional language in response to the US elections produced either by tweets celebrating Trump’s victory or mourning Clinton’s loss. Study 2 addressed the limitation introduced by the fact that both negative and positive tweets were taken in response to the same contentious context of the US 2016 elections. In this study, we examined data from two separate political situations, the celebration following the supreme court ruling for same-sex marriage and the Ferguson unrest. Based on the literature, we predicted that in predominantly negative situations, content containing negative emotional language would be shared more than content using positive emotional language. However, the conflicting theories and evidence did not justify any clear prediction about whether positive or negative language would spread more when the precipitating situation was positive. Analysis scripts and data are available at https://osf.io/xqevy.

## Study 1: Spread of Emotional Language for Users with Positive or Negative Evaluations of the Same Situation

In Study 1, we compared the spread of positive and negative emotional language in tweets that were produced in response to the results of the 2016 US presidential election. We analysed hashtags that were either created to celebrate Donald Trump’s victory (positive context) or to mourn Hillary Clinton’s loss (negative context).

### Method

#### Participants

We collected data from Twitter using GNIP (gnip.com), which allowed the download of full archives of tweets according to a specified word or hashtag. We focused on tweets of users mourning Hillary Clinton’s defeat and users celebrating Donald Trump’s victory in the 2016 US presidential elections. We chose to focus on post-election responses, because the election was frequently discussed online by supporters of both parties, who used distinctive hashtags depending on whether their candidate won or lost. We used specific hashtags to identify tweets, because previous research suggested that this method should lead to tweets that are more directly related to the situation and that mostly included users who support the specific cause (Barberá et al., 2015; Tufekci, 2014). The hashtags used to identify tweets mourning Hillary Clinton’s election defeat were: #TrumpRiot, #TrumpProtest, #ProtestTrump, #NotMyPresident, #HesNotMyPresident and #AntiTrump, and the hashtags used to celebrate Donald Trump’s election victory were #PresidentTrump, #TrumpWinner, #MakeAmericaGreatAgain, #MAGA, #TrumpWon and #Trump2020. We used the size of the dataset in Brady et al. (2017) as reference to the minimum amount of tweets required for data collection, setting 100,000 tweets as our minimum data sample with the hope of collecting a greater number of tweets that would permit the detection of even smaller effects. The data collection period was 7 days starting from the date of the election result. This time period was chosen to make sure that the hashtags were not utilized to discuss topics other than the specified situation. We collected only original tweets, which are tweets that users created themselves rather than those that included or shared a previous tweet. We also did not include replies to other users’ content to reduce the chance that the emotional language detected was actually in response to some original tweet. We also collected tweets of non-verified users in order to exclude celebrities, companies or other organizations whose behaviour has been found to differ significantly from normal users (Brady et al., 2017). Finally, we restricted our dataset to tweets that were written in English. The total number of tweets mourning Hillary Clinton’s defeat was 346,730, produced by 161,194 individual users (29.88% of users wrote multiple tweets) and 278,188 tweets celebrating Donald Trump’s victory from 82,970 individual users (28.42% of users wrote multiple tweets). An updated number of shares for each original tweet were collected in a second data collection effort using the Twitter API. As some of these posts were deleted by the users who created them, it is important to note that this sample described above only includes tweets that were still accessible with the API in 2019.

### Measures

We used the sentiment analysis classifier SentiStrength (Thelwall et al., 2010) to evaluate the intensity of positive and negative emotional language used in each tweet. SentiStrength is especially suitable for short texts such as tweets due to its capability to integrate syntactic rules such as negation, amplification and reduction, to interpret repetition of letters and exclamation points as expressing stronger emotions and to capture emoticons (Ribeiro et al., 2016). For each individual tweet, SentiStrength provides a score of positive and negative emotional intensity ranging from 1 (neutral) to 5 (intense). Each number represents the intensity of negative and positive emotional language, allowing us to capture mixed emotional language within a single tweet. We subtracted the emotional language intensity scale by one to make it easier to interpret the intercepts of the regression models, thus creating 0–4 scales. To make sure that SentiStrength results were consistent with other sentiment analysis tools, we correlated the values produced by SentiStrength to VADER (Gilbert & Hutto, 2014), which is another sentiment analysis tool that have been found to be especially useful in the analysis of short texts. The correlation between VADER and SentiStrength was between r = 0.52–0.62 in our datasets (see Supplementary Material for more detailed analysis of the datasets using VADER).

As the dependent variable, we measured the number of shares of the original tweet. We do not report how emotional language influenced likes in the main manuscript, as likes are often used to signal a variety of reactions other than mere support. However, results from this analysis are reported in the Supplementary Materials.

#### Results

##### Valence of the Situation

Our first analysis was designed to confirm that the two selected situations were indeed correctly categorized as either positive or negative by checking that the tweets in response to a situation used more positive or negative emotional language. We used a linear mixed model to compare the intensity of positive and negative emotional language score for each situation. The independent variable was the valence category produced by the sentiment analysis (positive or negative). Our dependent variable was the intensity score corresponding to each valence. As some participants wrote more than one tweet, we added a random intercept of user ID to the model. For the tweets mourning Hillary Clinton’s election loss, the mean score for negative emotional language was significantly higher than the mean score for positive emotional language (*b* =  − 0.56 [0.55, 0.56], *SE* = 0.002, *t* (599,528.76) =  − 261.7, *p* < 0.001, *R*^2^ = 0.085^1^), verifying that the situation was predominantly negative. For tweets celebrating Donald Trump’s victory, the mean score for positive emotional language in the tweets was significantly higher than the mean score for negative emotional language (*b* = 0.019 [0.015, 0.023], *SE* = 0.002, *t* (491,557.65) = 8.38, *p* < 0.001, *R*^2^ = 0.00012), verifying that the situation was predominantly positive. However, it is important to note that the difference between positive and negative scores was much smaller than in the negative situation, which was another reason for our decision to replicate these results in another context.

##### Predicting the Spread of Emotional Language

Our analysis was designed to assess the degree to which positive and negative emotional language scores predicted the spread of content. For both situations, we conducted linear mixed model analysis predicting the number of retweets using both the positive and the negative emotional language score for each tweet, as well as their potential interaction. The interaction term was introduced to the model in order to assess whether the associate between the increases of one type of valence was especially strong in relation to the other valence. As many tweets did not receive any retweets, the distribution of retweets was positively skewered around zero. We tested a few methods to account for this skewed distribution, all generating more or less similar results (for full analysis of all models, see Supplementary Materials). The most predictive model out of all examined transformations was a reciprocal transformation model, denoted by f(x) = 1 − (x + 1)^-1^. In principle, a reciprocal transformation involves raising x by the power of minus one. However, since many of our retweet numbers were zeros, we first conducted a x + 1 transformation. Finally, while reciprocal transformation reduces the skewness in the data, it also reveres the numbers’ magnitude. To account for this, we subtracted the number from 1, which was the largest number after the transformation. We then conduced a mixed model analysis predicting the transformed number of retweets by the positive and negative language scores as well as their interaction. We also included the user’s number of followers as a covariate, because users with more followers generally have more retweets regardless of the emotional content of their tweets. Finally, we added a random intercept of user ID to the model, as some users wrote more than one tweet. Starting with the results for the tweets mourning Hillary Clinton’s election loss, the negative language intensity of tweets was associated with an increased number of retweets (*b* = 0.0060 [0.0049, 0.0070], *SE* = 0.00045, *t* (341,547.25) = 13.45, *p* < 0.001, *R*^2^ = 0.073), whereas the positive language intensity of tweets was not significantly associated with the number of retweets (*b* = 0.0010 [− 0.00077, 0.0023], *SE* = 0.00080, *t* (339,386.68) = 1.25, *p* = 0.209, *R*^2^ = 0.073). The interaction between positive and negative emotional language and number of retweets was not significant (*b* = 0.00065 -0.00049, 0.0020], *SE* = 0.00054, *t* (336,629.82) = 1.21, *p* = 0.225, *R*^2^ = 0.073).

**Fig. 1:**
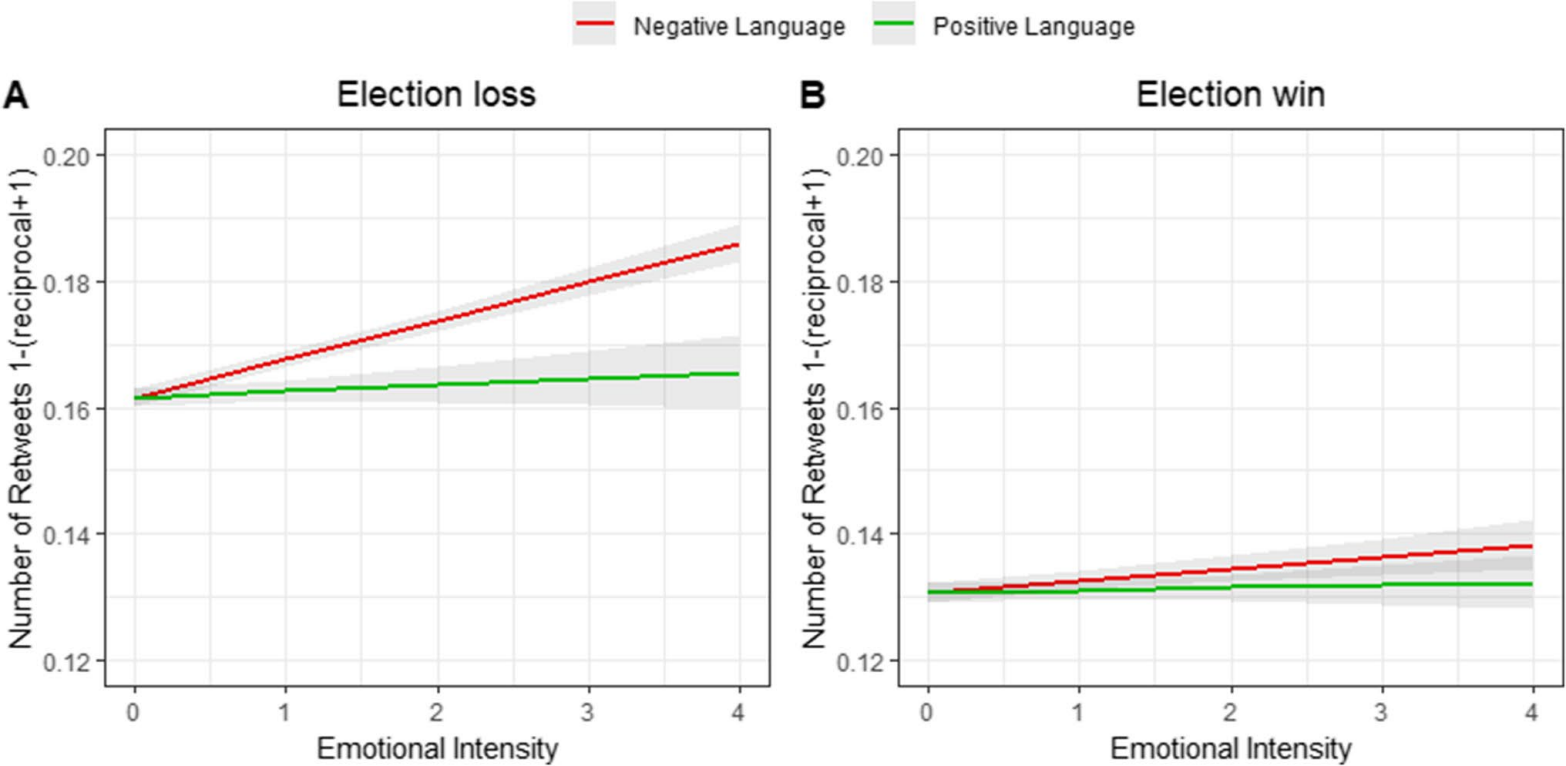
Results from Study 1 of the degree of emotional language intensity (negative and positive) predicting the number of retweets 1-(reciprocal + 1) transformed. For both sets of tweets (**A**), we found that an increase in negative language intensity was associated with an increase in the number of retweets, while positive language was not associated with the number of retweets. For the tweets celebrating Donald Trump’s victory (**B**), we again found that higher negative language scores were associated with higher number of retweets. Positive language intensity was not associated with the number of retweets

We then turned to the positive context of tweets celebrating Donald Trump’s victory. In this context as well, increased negative language intensity predicted the number of retweets (*b* = 0.0018 [0.00053, 0.0027], *SE* = 0.00054, *t* (271,470.69) = 3.44, *p* < 0.001, *R*^2^ = 0.11), whereas positive language intensity was not significantly associated with the number of retweets (*b* = 0.00039 [− 0.00095, 0.0016], *SE* = 0.00062, *t* (277,301.19) = 0.63, *p* = 0.52, *R*^2^ = 0.11, Fig. 1). The interaction between positive and negative emotional language and the number of retweets was again not significant (*b* =  − 0.00068 [− 0.0016, 0.00049], *SE* = 0.00055, *t* (270,670.85) =  − 1.22, *p* = 0.21, *R*^2^ = 0.11).

##### Political Affiliation of Users Producing Negative Tweets

The fact that the use of negative emotional language predicted more retweets, even for the tweets that celebrated Trump’s victory, called for further analysis of the users’ political affiliation. Most importantly, it was necessary to make sure that negative tweets in this positive context were mainly written by supporters of the victorious candidate (conservatives), rather than opponents (liberals). If it was mainly Hillary Clinton supporters who used negative language in the tweets that were categorized as celebrating Donald Trump’s victory, the increased spread of these tweets would capture intergroup dynamics rather than intragroup ones. Although it is well established that the content produced on social media is mainly driven by intragroup dynamics in echo-chambers of like-minded users (Bail et al., 2018; Boutyline & Willer, 2017; Brady et al., 2019), it is possible that the specific context of the election introduced intergroup dynamics too. We assessed this possibility by estimating users’ political affiliations.

Previous research has suggested that political affiliation can be predicted by determining which political figures users follow (Bail et al., 2018; Barberá, 2015; Brady et al., 2017; Goldenberg et al., 2020). Thus, users who follow a greater number of liberal political figures are more likely to be liberals (after excluding extremely popular politicians such as the president or candidates who are running for office from the count) (Barberá, 2015). We used the same approach to estimate users’ political affiliation by utilizing Bail et al. (2018) list that contained 4,176 public figures and organizations. To further understand the spread of negative language that was written using the hashtags that celebrated Trump’s victory, we tested a subsample of posts (30,830 tweets) that were negative (minimum 2 on a scale to 4 in negative intensity) and had at least one retweet. We downloaded all these users’ follower lists using the Twitter API and counted how many of the followed accounts were more associated with conservatives (1,602 figures) and liberals (2,574 figures) from Bail et al.’s list. We categorized a user as conservative if they followed more Twitter accounts that were associated with conservatives on the list and as liberals if they followed at least as many figures associated with liberals (thus adopting a more statistically conservative measure of political affiliation). Using the method specified above, we were able to estimate the political affiliation for 85.9% of the users we tested. Of the sample of 30,830 tweets. 73.44% (22,642) were from conservatives, while the remaining 26.55% (8,188) were from liberals. In other words, our political affiliation classification suggested that the tweets that used negative language in the positive context were produced primarily by conservatives. Following our political affiliation analysis, we repeated our primary analysis of predicting shares from positive and negative emotional language, this time looking only on the subsample of users that we identified as conservatives. This analysis led to the same results as the more inclusive analysis (see Supplementary Materials for full description).

##### Analysis of Content of Negative Tweets

A second question related to the content of the language used in the negative tweets that were produced in the positive context. To investigate the content of these tweets, we conducted topic modelling using Latent Dirichlet Allocation (LDA) (Berger & Packard, 2018; Blei, 2012). This method takes texts and estimates latent topics and themes discussed in the texts by measuring word co-occurrence within and across documents (Berger & Packard, 2018). Documents for topic modelling can be a collection of texts such as a chapter of a book, newspaper articles or a collection of tweets such as this example. Using this method allowed us to (1) assess if the topics of positive and negative tweets differed from each other and (2) to qualitatively evaluate the topics that were unique in each of these two groups.

To achieve these two goals, we first classified each tweet as positive or negative on the basis of whether it had a higher positive or negative SentiStrength score (excluding tweets with equal positive and negative scores). We used the “topicsmodel” package to conduct the topic modelling analysis on RStudio (Grün et al., 2020). The number of possible topics was manually pre-defined to two in an attempt to capture the two categories of positive and negative emotional languages and thus to maximize interpretability of the model (Jacobi et al., 2016). These topic models were generated by calculating and by measuring the co-occurrence of single words (unigrams) and two-word phrases (bigrams).

Our analysis focused on tweets celebrating Donald Trump’s victory, as we were primarily interested in the topics that were addressed in the positive context. We investigated if the difference of the topics based on their calculated valence (positive and negative tweet) by analysing if one topic was predominantly represented in one of the categories and not in the other. A γ-value represents how much a category is associated with a certain topic (per-document topic probability, for an overview, see Silge and Robinson (2017)). In this example a high γ-value means that the words appearing within a category (positive or negative) have a higher probability to only be used in one of the topics, meaning that there are only a few terms that occur often in both categories (Kee et al., 2019). Looking first at the positive tweet subcategory, we found it was mostly associated with the first topic detected by topic modelling. The proportion of words in the positive tweet category that are more likely coming from the first topic was γ > 99.9% in the uni- and bigram analyses. The negative category was mainly associated with the second topic detected by topic modelling (γ > 99.9%) for the uni- and bigram analysis. This meant that results from the sentiment analysis were congruent with the topic modelling analysis such that the topics in the two valenced categories of tweets (positive, negative) were distinguishable.

To understand the content of each topic, we examined the unigrams and bigrams that were more likely to be used in one topic but unlikely to be used in the other. The likelihood of a uni- or bigram being used in a specific topic is defined as a beta score β (“beta-scores”). While the words that are most frequent in a topic overlap to a certain extent, comparing words and two-word phrases that are unique to each topic helps to identify the nature of the topic and its underlying content. To do this, we identified the unigrams and bigrams with the greatest differences between the β-values of positive and negative tweets using log ratios (Silge & Robinson, 2017): log_2_($\frac{{\beta }1}{{\beta }2}$). Table 1 shows the top 5 most indicative uni- and bigrams for each topic. The most indicative unigrams and bigrams for the positive tweets highlighted the happiness and pride of supporters of the winning party. The hashtag “#TCOT” (“top conservatives on twitter”) was the most indicative unigram for the negative tweets. TCOT is used by conservatives to locate and identify tweets of other conservative users, which again confirms that these tweets were probably produced by conservatives. The other unigrams seemed to be directed towards opponents such as liberals or the media. The next common distinctive words were “lies” and “hate” which could possibly indicate the troubles that this group had to endure in order to achieve their victory. The most indicative bigram for the negative tweets was the conservative phrase “USA Trumptrain”, again suggesting that these tweets were created by conservatives. The other bigrams highlight the still ongoing conflict by talking about “trump war” or even going as far as calling this conflict a second civil war. We also added the top 10 tweets with the most number of retweets from the negative category to the Supplementary Material to show some examples of these viral negative language tweets.

**Table 1:** The 5 most unique uni- and bigrams for each topic. This table shows the words showing the biggest differences in log_2_($\frac{{\beta }1}{{\beta }2}$) when comparing positive and the negative tweets ordered by the size of this difference (from top to bottom). Topic 1 shows those with higher log_2_($\frac{{\beta }1}{{\beta }2}$) values for positive tweets and topic 2 shows words with higher absolute log_2_($\frac{{\beta }1}{{\beta }2}$) values for negative tweets in the context of celebrating Donald Trump’s election victory

Topic 1 (positive tweets):				Topic 2 (negative tweets)			
Unigrams	log_2_($\frac{{\beta }1}{{\beta }2}$)	Bigrams	log_2_($\frac{{\beta }1}{{\beta }2}$)	Unigram	log_2_($\frac{{\beta }1}{{\beta }2}$)	Bigrams	log_2_($\frac{{\beta }1}{{\beta }2}$)
Hope	151.11	Wow election night	129.53	#tcot	− 221.24	USA trumptrain	-134.95
Donald	148.22	Reince MAGA	128.17	Liberal	− 220.56	War trump	-134.40
Love	147.41	Sweet patriot	127.68	Media	− 219.82	Beat Hillary	-133.46
Happy	147.39	Beautiful baby	127.22	Lie	− 219.32	Time ago	-133.21
Awesome	147.39	Follow god	126.10	Hate	− 219.26	Civil war 2	-133.02

To summarize, results in Study 1 indicated that increased use of negative language was associated with a greater number of retweets in both positive and negative situations, whereas increased use of positive language was not associated with the numbers of retweets. Analysis of users’ political affiliation indicated that the negative tweets that were produced under the context of celebrating Donald Trump’s victory were produced mostly by conservatives. Further analysis of the negative texts in this context suggested that the most commonly unique words in the negative were used to discuss potential obstacles and adversaries in the path to victory.

## Study 2: Separate Positive and Negative Situations

One limitation of Study 1 was that it examined the spread of emotional language in the context of the 2016 US presidential elections which were one the most contentious political events in the past decade, filled with intergroup conflicts and hostility. A second limitation of Study 1 was that the degree of positivity in the positive event was not as strong as the degree of negativity in the negative event. In Study 2, we were hoping to address these limitations by choosing a political celebration that involved less conflict between groups and in which the events were more clearly negative and positive. Additionally, since the users sampled for the positive context in Study 1 were predominantly conservatives, we were hoping to replicate the result by looking at a political event that was celebrated primarily by liberals. Therefore, in Study 2, we compared two separate political events, one negative and one positive. The negative situation was the Ferguson unrest following the police shooting in 2014 of Michael Brown, and the positive situation was the 2015 US Supreme Court ruling approving same-sex marriage.

### Method

#### Participants

As in Study 1, we conducted a hashtag search via GNIP to collect data. The negative situation was the unrest in Ferguson in response to the killing of the black teenager Michael Brown on August 9th, 2014, and the positive situation was the US Supreme Court’s ruling in favour of same-sex marriage on the 26th of June 2015. Our criteria for the selection of these situations were that they were both frequently discussed, distinct (thus removing the possibility of tweets that covered both situations) and specifically identifiable using Twitter hashtags. Again, we only downloaded original tweets (tweets with new content) and did not include replies, retweets or tweets from verified accounts. Tweets were collected for 7 days following the elicitation of each event. The hashtags used to identify tweets related to the Ferguson unrest were #Ferguson, #MichaelBrown, #MikeBrown, #Blacklivesmatter and #raceriotsUSA,and the hashtags used to identity tweets related to the same-sex marriage ruling were #LoveWins, #LoveisLove, #SameLove, #LGBT, #SCOTUSMarriage and #MarriageEquality. The dataset for the Ferguson unrest dataset included 552,911 original tweets from 181,179 individual users (39.47% of users wrote multiple tweets), and the same-sex marriage ruling included 571,376 tweets from 389,111 individual users (31,89% of users wrote multiple tweets).

### Measures

As in Study 1, we used SentiStrength to analyse the tweets’ emotional language and counted the number of shares as our dependent variable.

#### Results

##### Valence of the Situation

As in Study 1, we estimated the predominant valence of the emotional language expressed in the tweets by comparing the positive and negative emotional language score of each situation. Results suggested that the positive emotional language score was significantly lower than the negative emotional language score for the Ferguson unrest tweets (*b* =  − 0.62 [− 0.61, − 0.63], *SE* = 0.001, *t* (1,004,347.25) =  − 387.10, *p* < 0.001, *R*^2^ = 0.13), whereas the positive emotional language score was significantly higher for the same-sex marriage tweets (*b* = 0.60 [0.61, 0.63], *SE* = 0.0015, *t* (968,932.36) = 390.3, *p* < 0.001, *R*^2^ = 0.12). Notice that unlike in Study 1, the size of the difference between positive and negative emotions in each context was similar.

##### Predicting Emotional Language Spread

Using a similar model to the one used in Study 1, we examined the degree to which positive and negative emotional language predicted retweets. Starting with the results for the Ferguson unrest, we found that the negativity of tweets was significantly associated with the number of retweets (*b* = 0.0079 [0.0071, 0.0088], *SE* = 0.00036, *t* (548,570.07) = 21.56, *p* < 0.001, *R*^*2*^ = 0.13), whereas the positivity of tweets was not a predictor of the number of retweets (*b* =  − 0.0011 [− 0.0024, 0.00041], *SE* = 0.00072, *t* (546,218.12) =  − 1.53, *p* = 0.12, *R*^*2*^ = . 13). There was no significant interaction between the effects of negativity and positivity on the number of retweets (*b* =  − 0.00054 [− 0.0017, 0.00037], *SE* = 0.00051, *t* (545,069.56) =  − 1.05, *p* = . 29, *R*^*2*^ = 0.13).

**Fig. 2:**
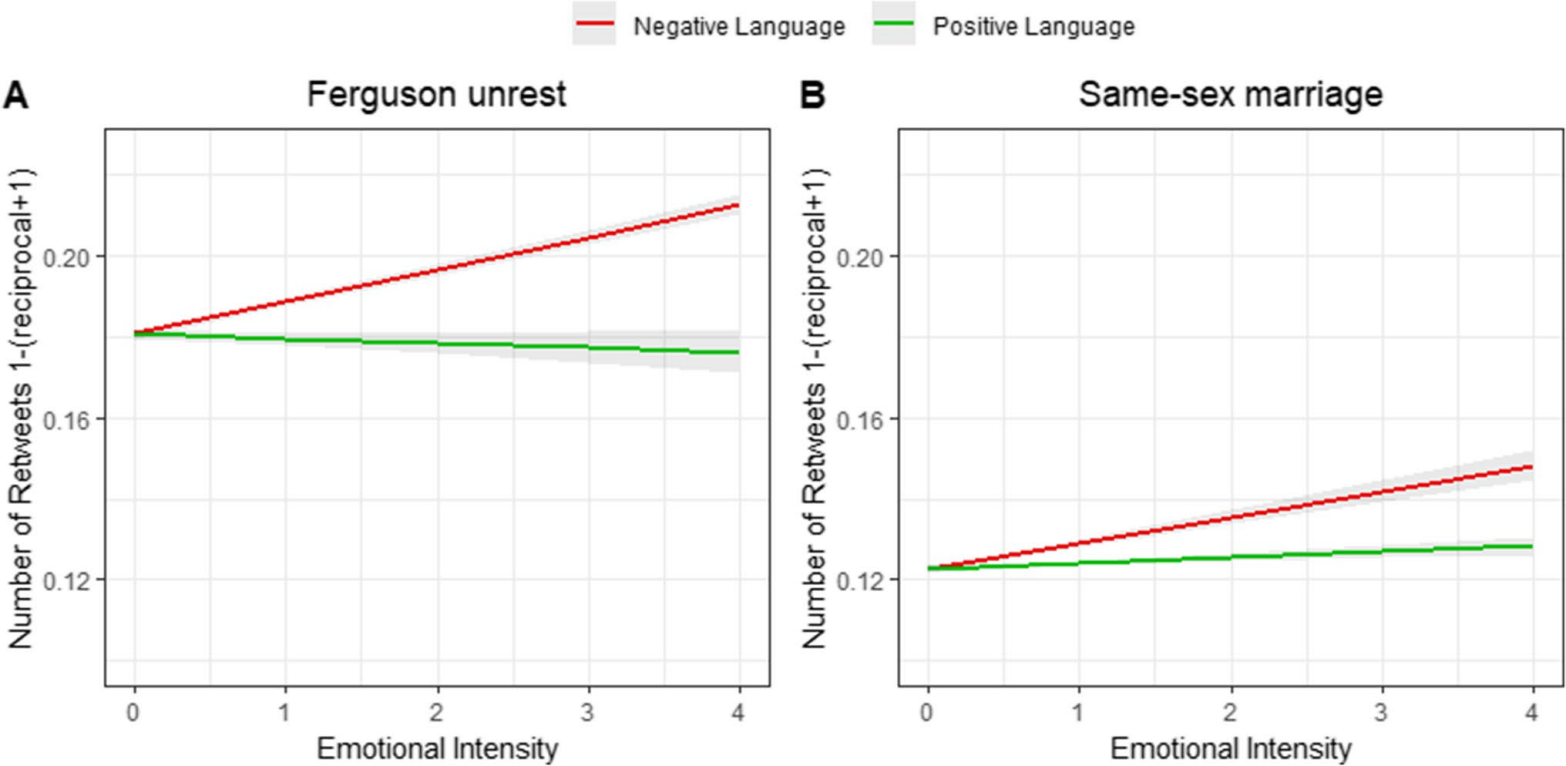
Results from Study 2 of emotional language intensity (negative and positive) predicting number of retweets (reciprocal + 1 transformed). For the Ferguson unrest (**A**), we found that an increase in negative language intensity was associated with an increase in the number of retweets, while an increase in positive language was associated with a decrease in retweets. For the same-sex marriage ruling (**B**), we again found that higher negative language scores were associated with higher number of retweets. Positive language intensity was also associated with the number of retweets in this context

For the tweets relating to the same-sex marriage ruling, the negativity of tweets was associated with that of the number of retweets (*b* = 0.0063 [0.0051, 0.0075], *SE* = 0.00051, t (548,203.04) = 12.43, *p* < 0.001, *R*^*2*^ = 0.16). The positivity of tweets was also a significant predictor of the number of retweets for the positive situation (*b* = 0.0014 [0.00085, 0.0020], *SE* = 0.00035, *t* (567,799.97) = 4.02, *p* < 0.001, *R*^*2*^ = 0.16, see Fig. 2). There was again no significant interaction between the effects of negativity and positivity on the number of retweets (*b* = 0.00057 [− 0.00012, 0.0015], *SE* = 0.00039, *t* (548,974.21) = 1.45, *p* = 0.14, *R*^*2*^ = 0.16). These results were similar to those of Study 1 with one difference: positive language predicted an increase in the number of shares in the positive situation but nonetheless with a smaller coefficient than for the negative language.

##### Political Affiliation of Users Producing Negative Tweets

As in Study 1, we evaluated the political affiliation of the users who wrote negative tweets in the positive context (same-sex marriage) to make sure that most of them were indeed liberals. We focused our analysis on users who wrote tweets that were at least moderately negative (scoring a minimum of 2 on the SentiStrength scale that has a maximum value of 4, *N* = 4,816). Using the method specified in Study 1, we were able to estimate the political affiliation of 92.11% of the users we tested. Of the sample, 94.45% (4.549) were identified as liberals, while the remaining 5.54% (267) were identified as conservatives. This political affiliation analysis suggested that the negative tweets produced in the positive context (same-sex marriage) were primarily written by liberals.

##### Analysis of Content of Negative Tweets

As in Study 1, we conducted topic modelling to estimate the content of the tweets that were produced in the positive context, using the same topic modelling method as in Study 1. We again found that there was one topic uniquely associated with the positive category and another topic uniquely associated with the negative category for both uni- and bigram analyses. This distinctiveness of categories was indicated by the γ-scores of > 99.9 each. In other words, the categorization based on topics modelling was in line with the sentiment analysis, thereby supporting the idea of two distinct categories of positive and negative tweets.

See Table 2 for 5 unigrams and bigrams that were most indicative for the two categories. The uniquely positive words seemed to describe the situation and its significance. The uniquely negative words, on the other hand, potentially captured some of the negative connotations of this victory for the supporters of the same-sex marriage ruling. The most common unigram, “tears”, is likely expressing tears of joy and signify positive emotion (see further analysis below to address this issue). Other frequent negative words in this category such as “fight” and “hate” were likely used to describe the challenging path that led to the ruling. The most unique negative two-word phrase (bigram) was “ignorant people” which again suggests that these tweets were indeed created by supporters and were used to express negative emotions towards the outgroup. Similarly, the bigram “terrible Christian” confirmed that some of the negative tweets were directed at the outgroup. However, we again found combinations including the word “tear” which could indicate a more complex emotional experience. To make sure that our results are not driven by terms like “tears”, “sobbing” and “cry” that likely represent positive emotions, we evaluated their contribution to the overall dataset. Looking at the amount of words that contained terms related to crying, we found that these words appeared in only 2.7% of tweets. We then removed these tweets from the database and ran the above analysis, again finding similar results (see Supplementary Materials for full description). We again added the top 10 tweets with the most number of retweets from the negative category to the Supplementary Material to show some examples of these viral negative language tweets.

**Table 2:** The 5 most unique uni- and bigrams for each topic. This table shows the words showing the biggest differences in log_2_($\frac{{\beta }1}{{\beta }2}$) when comparing positive and the negative tweets ordered by the size of this difference (from top to bottom). Topic 1 shows those with higher log_2_($\frac{{\beta }1}{{\beta }2}$) values for positive tweets and topic 2 shows words with higher absolute log_2_($\frac{{\beta }1}{{\beta }2}$) values for negative tweets in the positive situation

Topic 1 (positive tweets):				Topic 2 (negative tweets)			
Unigrams	log_2_($\frac{{\beta }1}{{\beta }2}$)	Bigrams	log_2_($\frac{{\beta }1}{{\beta }2}$)	Unigram	log_2_($\frac{{\beta }1}{{\beta }2}$)	Bigrams	log_2_($\frac{{\beta }1}{{\beta }2}$)
Week	44.31	Marriage amaze	48.49	Tear	− 161.00	Ignorant ppl	− 257.25
Rule	42.49	Magnificent day	46.49	Watch	− 160.33	Tear finally	− 254.99
Life	41.91	Perfect love	46.36	Hate	− 160.08	Terrible Christian	− 253.86
Decision	41.43	Equal marriage love	45.98	Fight	− 159.75	Marriage dissent	− 253.56
Nationwide	41.14	Beautiful	45.69	They’re	− 158.94	Tear marriage equality	− 253.07

To summarize, the results of Study 2 replicated those of Study 1, suggesting that the use of negative language was associated with a greater number of retweets in both the positive and negative situations. The use of positive language was associated with an increase in the number of retweets only in the context of the positive situation. To alleviate the concern that the spread of negative language following the positive situation arose from opponents to same-sex marriage, we conducted additional analysis confirming that likely supporters of same-sex marriage in fact produced most of the negative content.

## General Discussion

The goal of this research was to investigate the types of emotional language that spread further in response to both negative and positive political situations. In Studies 1 (same situation) and 2 (separate situations), we examined the spread of emotional language in response to predominantly negative and positive situations. Results from both of our studies suggested that negative language tended to spread further both in negative and positive situations. Analysis of political affiliation in both studies indicated that the users that produced the negative language in the political celebrations were mainly ingroup members (conservatives in Study 1 and liberals in Study 2). Analysis of the content of the negative language that were written in response to the celebrations also showed that negative language was mainly used to describe hardships and past obstacles or to describe the outgroup in a negative way. Combined, these two studies shed light on the nature of political engagement online.

### Implications of the Findings

The fact that even in political celebrations negative tweets were likely to be shared more sheds light on the affective nature of political interactions on social media. This is especially troubling because interactions on social media have a reinforcing nature, such that users are likely to produce content that is similar to previous content that generated engagement (Brady & Crockett, 2019; Brady et al., 2019). Increased retweeting of content containing negative language is therefore likely to lead users to further produce more such content and perpetuate the use of negative language (Brady et al., 2021).

Users’ preference for negative content, even in political celebrations, is in line with the idea that intergroup context makes negative emotions more prevalent (Cikara, 2015). Assuming that negative language is shared more frequently, and considering the fact that a portion of this language, even in political celebrations, is used to express feelings towards rival groups, these dynamics are likely to perpetuate more intergroup hostility, which plays a role in affective polarization and sectarianism (Finkel et al., 2020; Iyengar et al., 2019).

The increase in negative language on social media may also affect viewers’ emotions and general well-being. It is not yet clear whether all forms of engagement with social media are associated with decreased well-being, since effects probably vary depending on both the situation and the person (Kross et al., 2020). However, engagement in political contexts, especially when they involve negative language, may be one of the situations in which negative effects are more likely to occur. For example, a longitudinal study demonstrated that daily exposure to political situations was associated with worse physical and mental well-being (Feinberg et al., 2020). Additionally, exposure to hateful content can decrease social trust (Näsi et al., 2015) and exaggerate the feeling of outgroup threat (Lees & Cikara, 2020), which could add to negative well-being outcomes. Further work should examine the consequence of engaging in political discourse online on users’ well-being.

### Limitations and Future Directions

Despite the intriguing findings and the measures we took to dispel alternative hypotheses, there are limitations to the studies that should be addressed in future research. One possible issue pertains to the question of whether negative emotional language is acutely used to express negative emotions. One especially important example is the word “tear” that was prevalent in the same-sex marriage ruling content and was categorized as negative while is likely reflecting positive emotions. It is first important to note that removing these words (which represented 2.7% of the tweets) did not change the direction or strength of the results. However, quantifying the problem of mapping emotional language to emotions is important. One way to examine this issue is by looking at papers that evaluated the quality of the sentiment analysis tools compared to human annotators. One example is Ribeiro et al.’s (2016) comparison of sentiment analysis tools, which found that in 90% of cases, SentiStrength produced the same emotional category as human annotators. However, larger datasets and improved tools could lead to a more fine-grained analysis of the language used in positive contexts and better understanding of the content used in these contexts.

A second limitation of the study is that our observational methodology did not permit us to directly assess the social and psychological mechanisms that stand at the base of the spread of negative language. In the introduction, we provided a few suggestions for potential mechanisms. We believe that the best way to test those mechanisms is to conduct behavioural experiments in which participants see the content produced by researchers and are asked to share posts that they like. Tracking users’ attention, emotional arousal and memory of these content may help us in further understanding the underlying mechanism.

A third limitation of the current paper is the possibility that much of the negative tweets that were produced in the positive context were actually parts of intergroup interactions that were not well captured by us. In order to minimize this problem, we focused only on original tweets and removed replies from the analysis. However, it is very hard to evaluate whether a negative tweet was written when citing an outgroup news source or indirectly responding to an outgroup user. Future work should use experiments to examine the degree to which negativity is elicited even in the absence of any content from outgroup member.

Fourth, one question is whether increased negativity is more likely to spread in political celebrations even in cultures in which high arousal negativity is less prevalent (Tsai, 2019). One initial answer to this question are results from a study by Fan and et al. (2016), which examined emotional contagion of positive and negative emotions in Weibo, which is a Chinese social network. Their results showed that anger spread further and faster than joy even in general interactions on Weibo, suggesting that the increased spread of negativity is cross-cultural. However, this study was done for general content, and further studies should examine the spread of emotions specifically in political context.

Finally, another limitation of the current project is that it examined only online interactions. Therefore, one questions is whether these findings could be generalized outside of social media. Although many argue that hostile intergroup dynamics are amplified on social media, either due to the nature of social interactions or by top-down algorithms promoting more emotional content (Goldenberg & Gross, 2020), it is likely to assume that these dynamics represent some general tendencies that people have. For example, although it is common to believe the political echo-chambers are especially prevent on social media, recent work has found that segregation based on physical location may even be greater (Brown & Enos, 2021). Further work should examine emotional dynamics as the unfold outside of social media.

Despite these limitations, we believe that our findings reveal an important aspect of political discourse on social media, pose interesting questions and open the door to future investigations and possibility even interventions designed to mitigate the spread of negative language.

## Supplementary Information

Below is the link to the electronic supplementary material.

Supplementary file1 (DOCX 384 KB)

## Footnotes

1 Marginal *R*^2^ calculated based on recommendations from Nakagawa, Johnson, and Schielzeth (2017) using r package “MuMln” (Barton & Barton, 2015).

## Additional Information

### Data Availability

Analysis scripts and data are available at https://osf.io/xqevy. Studies were not preregistered.
