# Supplementary material for: Negativity Spreads More than Positivity on Twitter After Both Positive and Negative Political Situations
Source: Affect Sci. 2021 Oct 12;2(4):379–90. doi: 10.1007/s42761-021-00057-7 (PMC9383030; doi:10.1007/s42761-021-00057-7)
Supplement: Supplementary file 1 — Supplementary file1 (DOCX 384 KB) [file 42761_2021_57_MOESM1_ESM.docx]

**Negativity Spreads More than Positivity on Twitter after both Positive and Negative Political Situations (Supplementary Material)**

Table of Contents

[I. Emotional language intensity predicting likes in Studies 1 and 2 2](#_Toc71124870)

[Trump election loss 2](#_Toc71124871)

[Trump election win 3](#_Toc71124872)

[Ferguson unrest 5](#_Toc71124873)

[Same-sex ruling 6](#_Toc71124874)

[II. Emotional language intensity predicting retweets in subsets for positive situations 7](#_Toc71124875)

[Trump election win (Subset 22,642 tweets from conservatives) 7](#_Toc71124876)

[Same-sex ruling (Subset 7.661 tweets from liberals) 9](#_Toc71124877)

[III. Emotional language intensity predicting retweets: Model Comparisons 10](#_Toc71124878)

[Trump election win 12](#_Toc71124879)

[Hillary election loss 16](#_Toc71124880)

[Same-sex ruling 20](#_Toc71124881)

[Ferguson unrest 24](#_Toc71124882)

[IV. Emotional language intensity predicting retweets: General Additive Models 28](#_Toc71124883)

[Trump election win 28](#_Toc71124884)

[Hillary election loss 30](#_Toc71124885)

[Same-sex marriage 31](#_Toc71124886)

[Ferguson unrest 33](#_Toc71124887)

[V. Emotional language intensity predicting retweets: Sentiment Tool Comparison 35](#_Toc71124888)

[Trump election win 35](#_Toc71124889)

[Hillary election loss 36](#_Toc71124890)

[Same-sex ruling 38](#_Toc71124891)

[Ferguson unrest 39](#_Toc71124892)

[VI. Analysis of most viral negative tweets in positive contexts 41](#_Toc71124893)

[Trump election win 41](#_Toc71124894)

[Same-sex ruling 41](#_Toc71124895)

[VII. Removing emotional ambiguous words from same-sex dataset 42](#_Toc71124896)

[Same-sex ruling 42](#_Toc71124897)

[Reference 44](#_Toc71124898)

# **Emotional language intensity predicting likes in Studies 1 and 2**

We assessed the degree to which positive and negative emotion scores predicted the spread of content as measured by the number of likes. For both studies, we conducted linear mixed model analysis predicting the number of likes by both the positive emotion score and the negative emotion score for each tweet as well as their interaction. As many tweets did not receive any retweets, the distribution of retweets was positively skewered around zero. We therefore did a reciprocal transformation denoted by f(x) = 1-(x+1)⁻¹. A reciprocal transformation involves dividing 1 by the retweet number. Since many of our between numbers were zeros, we first conducted a x+1 transformation. Finally, while reciprocal transformation reduces the skewness in the data it also reveres the numbers’ magnitude. To account for this, we subtracted the number from 1, which was the largest number after the transformation. It is noteworthy that the distribution is still not strictly normally distributed due to the excess zeros. We again added a random intercept of user id to the model to deal with the issue of multiple tweets from the same users. We also included the user’s number of followers as a covariate, because users with more followers generally have more likes regardless of the emotional content of their tweets.

## Trump election loss

**Table 1S.** Linear mixed model with four factors (positive language, negative language, number of followers, positive language x negative language) and number of likes 1-(reciprocal+1) as the dependent variable.

| **Fixed Effects** | | | | | |
| --- | --- | --- | --- | --- | --- |
|  | Estimate | *SE* | 95% CI | *t* | *p* |
| Intercept | 0.29 | 0.00088 | 0.29 – 0.30 | 337.33 | .000 |
| Positive Language | 0.0093 | 0.00090 | 0.0075 – 0.011 | 10.32 | .000 |
| Negative Language | 0.0124 | 0.00050 | 0.011 – 0.013 | 24.45 | .000 |
| Number of followers | 0.077 | 0.00068 | 0.076 – 0.079 | 112.69 | .000 |
| Positive Language × Negative Language | -0.00088 | 0.00060 | -0.0020 – 0.00020 | -1.46 | .144 |
| **Random Effects** | | | | | |
|  | |  | Variance | *SD* |  |
| Participant (Intercept) | |  | 0.028 | 0.17 |  |
| Residual | |  | 0.057 | 0.24 |  |
| **Model Fit** | | | | | |
| *R*^2^ | |  | Marginal | Conditional | |
|  | |  | 0.066 | 0.38 | |
| Model equation: Likes 1-(reciprocal+1) ~ Positive * Negative + centered (Followers) + (1 \| User) | | | | | |

*Notes.* Model fit was calculated using the R package MuMIn (Barton, 2018) based on the paper of Nakagawa et al. (2017).


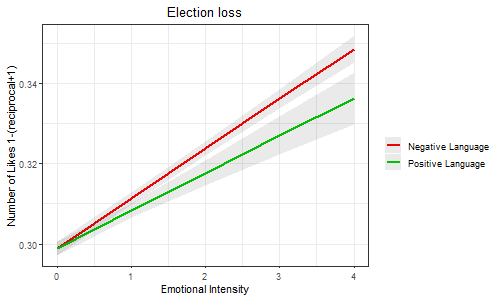


**Figure 1S.** Results of emotional language intensity (negative and positive) predicting number of likes (reciprocal+1 transformed) for the tweets mourning Hillary Clinton’s election loss. We found that an increase in negative language intensity was associated with an increase in the number of likes. Positive language intensity was also associated with an increase in likes.

## Trump election win

**Table 2S.** Linear mixed model with four factors (positive language, negative language, number of followers, positive language x negative language) and number of likes 1-(reciprocal+1) as the dependent variable.

| **Fixed Effects** | | | | | |
| --- | --- | --- | --- | --- | --- |
|  | Estimate | *SE* | 95% CI | *t* | *p* |
| Intercept | 0.27 | 0.00098 | 0.27 – 0.27 | 276.47 | .000 |
| Positive Language | 0.0071 | 0.00074 | 0.0057 – 0.0085 | 9.61 | .000 |
| Negative Language | -0.0015 | 0.00064 | -0.0027 – -0.00030 | -2.34 | .019 |
| Number of followers | 0.097 | 0.00085 | 0.095 – 0.098 | 113.87 | .000 |
| Positive Language × Negative Language | -0.00071 | 0.00065 | -0.0017 – 0.00049 | -1.07 | .280 |
| **Random Effects** | | | | | |
|  | |  | Variance | *SD* |  |
| Participant (Intercept) | |  | 0.038 | 0.20 |  |
| Residual | |  | 0.044 | 0.21 |  |
| **Model fit** | | | | | |
| *R*^2^ | |  | Marginal | Conditional | |
|  | |  | 0.10 | 0.52 | |
| Model equation: Likes 1-(reciprocal+1) ~ Positive * Negative + centered (Followers) + (1 \| User) | | | | | |

*Notes.* Model fit was calculated using the R package MuMIn (Barton, 2018) based on the paper of Nakagawa et al. (2017).


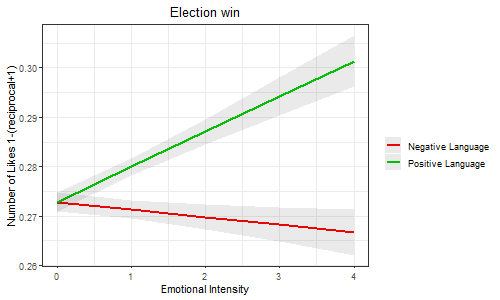


**Figure 2S.** Results of emotional language intensity (negative and positive) predicting number of likes (reciprocal+1 transformed) expressed in the tweets celebrating Donald Trump’s victory. We found that an increase in negative language intensity was associated with a decrease in the number of retweets (only example where negative language was a negative predictor), while an increase in positive language was associated with an increase in retweets. This result highlights the difference between likes and retweets as negative language was consistently a positive predictor for an increase in number of retweets, while we saw a decrease in the number of likes in this particular event.

## Ferguson unrest

**Table 3S.** Linear mixed model with four factors (positive language, negative language, number of followers, positive language x negative language) and number of likes 1-(reciprocal+1) as the dependent variable.

| Fixed Effects | | | | | |
| --- | --- | --- | --- | --- | --- |
|  | Estimate | *SE* | 95% CI | *t* | *p* |
| Intercept | 0.17 | 0.00066 | 0.17 – 0.18 | 277.83 | .000 |
| Positive Language | 0.013 | 0.00071 | 0.012 – 0.014 | 19.77 | .000 |
| Negative Language | 0.0096 | 0.00036 | 0.0087 – 0.010 | 26.76 | .000 |
| Number of followers | 0.079 | 0.00050 | 0.078 – 0.080 | 156.33 | .000 |
| Positive Language × Negative Language | -0.0017 | 0.00050 | -0.0029 – 0.00082 | -3.45 | .000 |
| Random Effects | | | | | |
|  | |  | Variance | *SD* |  |
| Participant (Intercept) | |  | 0.013 | 0.12 |  |
| Residual | |  | 0.051 | 0.22 |  |
| Model fit | | | | | |
| *R*^2^ | |  | Marginal | Conditional | |
|  | |  | 0.089 | 0.28 | |
| Model equation: Likes 1-(reciprocal+1) ~ Positive * Negative + centered (Followers) + (1 \| User) | | | | | |

*Notes.* Model fit was calculated using the R package MuMIn (Barton, 2018) based on the paper of Nakagawa et al. (2017).


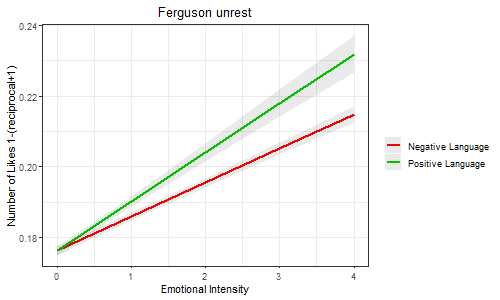


**Figure 3S.** Results of emotional language intensity (negative and positive) predicting number of likes (reciprocal+1 transformed) expressed in the Ferguson unrest tweets. We found that an increase in negative language intensity was associated with an increase in the number of likes.

## Same-sex ruling

**Table 4S.** Linear mixed model with four factors (positive language, negative language, number of followers, positive language x negative language) and number of likes 1-(reciprocal+1) as the dependent variable.

| Fixed Effects | | | | | |
| --- | --- | --- | --- | --- | --- |
|  | Estimate | *SE* | 95% CI | *t* | *p* |
| Intercept | 0.24 | 0.00064 | 0.23 – 0.23 | 381.09 | .000 |
| Positive Language | 0.0077 | 0.00044 | 0.0052 – 0.0070 | 17.40 | .000 |
| Negative Language | 0.0086 | 0.00063 | 0.0055 – 0.0085 | 13.64 | .000 |
| Number of followers | 0.098 | 0.00045 | 0.094 – 0.099 | 218.21 | .000 |
| Positive Language × Negative Language | 0.0024 | 0.00048 | -0.0012 – 0.0034 | 5.02 | .000 |
| Random Effects | | | | | |
|  | |  | Variance | *SD* |  |
| Participant (Intercept) | |  | 0.031 | 0.17 |  |
| Residual | |  | 0.047 | 0.21 |  |
| Model fit | | | | | |
| *R*^2^ | |  | Marginal | Conditional | |
|  | |  | 0.11 | 0.46 | |
| Model equation: Likes 1-(reciprocal+1) ~ Positive * Negative + centered (Followers) + (1 \| User) | | | | | |

*Notes.* Model fit was calculated using the R package MuMIn (Barton, 2018) based on the paper of Nakagawa et al. (2017).


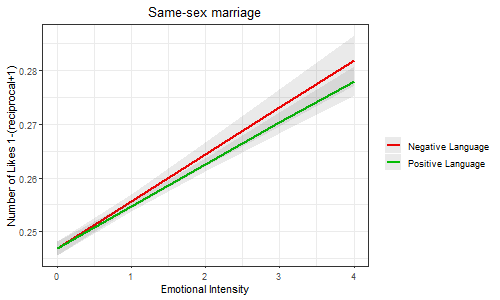


**Figure 4S.** Results of emotional language intensity (negative and positive) predicting number of likes (reciprocal+1 transformed) expressed in the same-sex marriage tweets. We found that an increase in negative language intensity was associated with an increase in the number of likes. Positive language intensity was also associated with an increase in likes.

# **Emotional language intensity predicting retweets in subsets for positive situations**

We repeated the main analysis of the manuscript that assessed the spread of content for both positive situations in each study (Study 1: Election win, Study 2: Same-sex marriage) with a subsample that only contained users that belonged to the political group that supported the event as estimated by our political affiliation estimation. This analysis addressed the concern that the effect of negative language on spreadibility of content was caused by an opposing political group expressing their negative language content.

## Trump election win (Subset 22,642 tweets from conservatives)

**Table 5S.** Linear mixed model with four factors (positive language, negative language, number of followers, positive language x negative language) and number of retweets 1-(reciprocal+1) as the dependent variable.

| Fixed Effects | | | | | |
| --- | --- | --- | --- | --- | --- |
|  | Estimate | *SE* | 95% CI | *t* | *p* |
| Intercept | 0.27 | 0.0042 | 0.27 – 0.28 | 66.40 | .000 |
| Positive Language | -0.0059 | 0.0026 | -0.010 – -0.0013 | -2.29 | .021 |
| Negative Language | 0.031 | 0.0020 | 0.026 – 0.035 | 15.24 | .000 |
| Number of followers | 0.071 | 0.0033 | 0.065 – 0.077 | 21.20 | .000 |
| Positive Language × Negative Language | 0.0059 | 0.0020 | 0.0025 – 0.010 | 2.89 | .003 |
| Random Effects | | | | | |
|  | |  | Variance | *SD* |  |
| Participant (Intercept) | |  | 0.40 | 0.63 |  |
| Residual | |  | 0.29 | 0.54 |  |
| Model fit | | | | | |
| *R*^2^ | |  | Marginal | Conditional | |
|  | |  | 0.071 | 0.40 | |
| Model equation: Retweets 1-(reciprocal+1)~ Positive * Negative + centered (Followers) + (1 \| User) | | | | | |

*Notes.* Model fit was calculated using the R package MuMIn (Barton, 2018) based on the paper of Nakagawa et al. (2017).


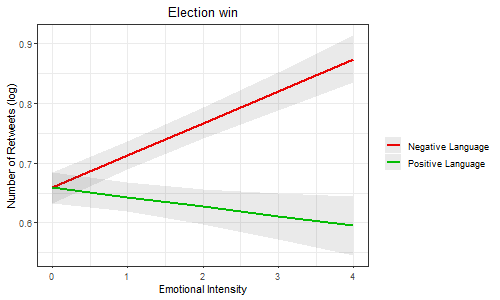


**Figure 5S.** Results of emotional language intensity (negative and positive) predicting number of retweets (1-(reciprocal+1) transformed) expressed in the tweets celebrating Donald Trump’s victory (subsample of estimated conservatives). We found that an increase in negative language intensity was associated with an increase in the number of retweets, while an increase in positive language was negatively correlated with the number of retweets.

## Same-sex ruling (Subset 7.661 tweets from liberals)

**Table 6S.** Linear mixed model with four factors (positive language, negative language, number of followers, positive language x negative language) and number of retweets 1-(reciprocal+1) as the dependent variable.

| Fixed Effects | | | | | |
| --- | --- | --- | --- | --- | --- |
|  | Estimate | *SE* | 95% CI | *t* | *p* |
| Intercept | 0.38 | 0.011 | 0.35 – 0.40 | 33.77 | .000 |
| Positive Language | 0.0026 | 0.0073 | -0.0091 – 0.017 | 0.35 | .723 |
| Negative Language | 0.060 | 0.0074 | 0.042 – 0.079 | 8.17 | .000 |
| Number of followers | 0.13 | 0.0083 | 0.12 – 0.15 | 16.25 | .000 |
| Positive Language × Negative Language | 0.0039 | 0.0056 | -0.0087 – 0.014 | 0.69 | .485 |
| Random Effects | | | | | |
|  | |  | Variance | *SD* |  |
| Participant (Intercept) | |  | 0.017 | 0.13 |  |
| Residual | |  | 0.052 | 0.22 |  |
| Model fit | | | | | |
| *R*^2^ | |  | Marginal | Conditional | |
|  | |  | 0.20 | 0.40 | |
| Model equation: Retweets 1-(reciprocal+1) ~ Positive * Negative + centered (Followers) + (1 \| User) | | | | | |

*Notes.* Model fit was calculated using the R package MuMIn (Barton, 2018) based on the paper of Nakagawa et al. (2017).


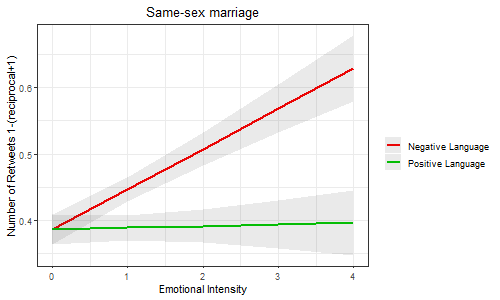


**Figure 6S.** Results of emotional language intensity (negative and positive) predicting number of retweets (1-(reciprocal+1) transformed) expressed in the same-sex marriage tweets (subsample of estimated liberals). We found that an increase in negative language intensity is associated with an increase in the number of retweets, while an increase in positive language was not correlated with number of retweets.

# **Emotional language intensity predicting retweets: Model Comparisons**

We tested three different models that could substitute the analysis presented in the manuscript. We compared (a) a linear models with 1-(reciprocal)+1 transformed retweet counts against (b) a linear models with log+1 transformed retweet counts (model from the manuscript), (c) a Poisson regression model, and (d) a negative binomial model. Based on the direct model comparison we decided to use the linear models with reciprocal+1 transformed retweet counts as it presented the best model fit in predicting retweets by the positive and negative sentiment of the text.

Model Details:

1. Linear regression 1-(reciprocal+1): This model is the one we originally presented in the paper. Our reciprocal transformation, is denoted by f(x) = 1-(x+1)⁻¹. In simple words, reciprocal transformation involves dividing 1 by the retweet number. Since many of our between numbers were zeros, we first conducted a x+1 transformation. Finally, while reciprocal transformation reduces the skewness in the data it also reveres the numbers’ magnitude. To account for this, we subtracted the number from 1. It is noteworthy that the distribution is still not strictly normally distributed due to the excess zeros. The model itself remained the same as in the manuscript meaning that we predicted the number of retweets using both the positive and the negative emotional language score for each tweet, as well as their interaction. The interaction term was introduced to the model in order to assess whether the effects of either content valence on spread depended on the level of the other content valence (thus testing for a possible effect of mixed emotional language). We again added a random intercept of user id to the model as some users wrote more than one tweet.

Lmer (reciprocal(retweet+1) ~

positive_score + negative_score + positive_score *negative_score + (user_id),

family = “gaussian”)

1. Linear regression log-transform: We log+1 transformed the number of retweets. The distribution was still severely skewed and had excess zeros, leading to a positive skew of the distribution. The model’s variables are identical to the ones from the previous model (a).

Lmer (log(retweet+1) ~

positive_score + negative_score + positive_score *negative_score + (user_id),

family = “gaussian”)

1. Poisson regression model: In an attempt to deal with the excess zeros we also considered a Poisson count model. The model’s variables were the same as previously specified in A.

Lmer (retweet ~

positive_score + negative_score + positive_score *negative_score + (user_id),

family = “Poisson”)

1. Negative binomial regression: Similar to the Poisson regression, we checked whether a negative binomial count model is better able to accommodate the large amount of zeros leading to a positive skew. The model’s variables are identical to the ones from the previous model (A.).

Lmer.nb (retweet ~

positive_score + negative_score + positive_score *negative_score + (user_id),

family = “negative binomial”)

Prediction of models: All models for all situations in the studies show similar results, finding that negativity spreads further than positivity. The only exception is the negative binomial model for the election win dataset, which did not show a significant association between positive and negative emotion scores of tweets and the number of retweets.

## Trump election win

**Table 7S. Linear regression 1-(reciprocal+1) model:** Linear mixed model with four factors (positive language, negative language, number of followers, positive language x negative language) and number of retweets 1-(reciprocal+1) as the dependent variable.

| Fixed Effects | | | | | |
| --- | --- | --- | --- | --- | --- |
|  | Estimate | *SE* | 95% CI | *t* | *p* |
| Intercept | 0.13 | 0.00077 | 0.13 – 0.13 | 167.80 | .000 |
| Positive Language | 0.0003 | 0.00062 | - 0.000811– 0.0016 | 0.63 | .52 |
| Negative Language | 0.0018 | 0.00054 | 0.00084 – 0.0028 | 3.44 | .000 |
| Number of followers | 0.0808 | 0.00064 | 0.079 – 0.082 | 124.61 | .000 |
| Positive Language × Negative Language | -0.00068 | 0.00055 | - 0.0019 – 0.00049 | -1.22 | .219 |
| Random Effects | | | | | |
|  | |  | Variance | SD |  |
| Participant (Intercept) | |  | 0.017 | 0.13 |  |
| Residual | |  | 0.034 | 0.18 |  |
| Model fit | | | | | |
| R^2^ | |  | Marginal | Conditional | |
|  | |  | 0.12 | 0.41 | |
| Model equation: Retweets 1-(reciprocal+1) ~ Positive * Negative + centered (Followers) + (1 \| User) | | | | | |

*Notes.* Model fit was calculated using the R package MuMIn (Barton, 2018) based on the paper of Nakagawa et al. (2017).

**Table 8S. Linear regression (log+1) model:** Linear mixed model with four factors (positive language, negative language, number of followers, positive language x negative language) and number of retweets (log+1) as the dependent variable.

| Fixed Effects | | | | | |
| --- | --- | --- | --- | --- | --- |
|  | Estimate | *SE* | 95% CI | *t* | *p* |
| Intercept | 0.21 | 0.0015 | 0.35 – 0.40 | 137.62 | .000 |
| Positive Language | 0.0024 | 0.0012 | -0.0052 – 0.0046 | 2.019 | .043 |
| Negative Language | 0.0046 | 0.0010 | 0.0023 – 0.0065 | 4.33 | .000 |
| Number of followers | 0.16 | 0.0013 | 0.16 – 0.17 | 124.28 | .000 |
| Positive Language × Negative Language | -0.0018 | 0.0010 | -0.0041– 0.00038 | -1.72 | .085 |
| Random Effects | | | | | |
|  | |  | Variance | *SD* |  |
| Participant (Intercept) | |  | 0.090 | 0.30 |  |
| Residual | |  | 0.12 | 0.35 |  |
| Model fit | | | | | |
| *R*^2^ | |  | Marginal | Conditional | |
|  | |  | 0.11 | 0.48 | |
| Model equation: Retweets (log+1) ~ Positive * Negative + centered (Followers) + (1 \| User) | | | | | |

*Notes.* Model fit was calculated using the R package MuMIn (Barton, 2018) based on the paper of Nakagawa et al. (2017).

**Table 9S. Poisson regression model:** Count mixed model with four factors (positive language, negative language, number of followers, positive language x negative language) and number of retweets as the dependent variable.

| Fixed Effects | | | | | |
| --- | --- | --- | --- | --- | --- |
|  | Estimate | *SE* | 95% CI | *t* | *p* |
| **Intercept** | -2.23 | 0.010 | -2.31– -2.16 | -213.30 | .000 |
| **Positive Language** | 0.15 | 0.0023 | -0.0010 – 0.0025 | 64.31 | .000 |
| **Negative Language** | 0.0084 | 0.0022 | -0.0045 – 0.019 | 3.74 | .000 |
| **Number of followers** | 0.92 | 0.0082 | 0.91 – 0.95 | 112.22 | .000 |
| **Positive Language x Negative Language** | -0.11 | 0.0024 | -0.016 – 0.0078 | -47.06 | .000 |
| Random Effects | | | | | |
|  | |  | Variance | *SD* |  |
| Participant (Intercept) | |  | 2.19 | 1.48 |  |
| Model fit | | | | | |
| *R*^2^ | |  | Marginal | Conditional | |
|  | |  | 0.09 | 0.33 | |
| Model equation: Retweets ~ Positive * Negative + centered (Followers) + (1 \| User), family = Poisson | | | | | |

*Notes.* Model fit was calculated using the R package MuMIn (Barton, 2018) based on the paper of Nakagawa et al. (2017).

**Table 10S. Negative binomial regression model:** Count mixed model with four factors (positive language, negative language, number of followers, positive language x negative language) and number of retweets as the dependent variable.

| Fixed Effects | | | | | |
| --- | --- | --- | --- | --- | --- |
|  | Estimate | *SE* | 95% CI | *t* | *p* |
| Intercept | -2.10 | 0.012 | -2.16 – -2.31 | -175.50 | .000 |
| Positive Language | 0.013 | 0.0069 | -0.010 – 0.025 | 1.86 | .616 |
| Negative Language | 0.0083 | 0.0060 | -0.045 – 0.019 | 1.37 | .168 |
| Number of followers | 0.95 | 0.0083 | 0.91 – 0.99 | 114.40 | .000 |
| Positive Language × Negative Language | -0.0056 | 0.0062 | -0.016 – 0.0078 | -0.905 | .365 |
| Random Effects | | | | | |
|  | |  | Variance | *SD* |  |
| Participant (Intercept) | |  | 1.88 | 1.37 |  |
| Model fit | | | | | |
| *R*^2^ | |  | Marginal | Conditional | |
|  | |  | 0.03 | 0.09 | |
| Model equation: Retweets ~ Positive * Negative + centered (Followers) + (1 \| User), family = Poisson | | | | | |

*Notes.* Model fit was calculated using the R package MuMIn (Barton, 2018) based on the paper of Nakagawa et al. (2017).

**Table 11S. Model Comparison:** Comparing log-likelihood scores of the above described models.

|  | Marginal *R*^2^ | AIC | BIC | Log-Likelihood | df | chi-squared | *p* |
| --- | --- | --- | --- | --- | --- | --- | --- |
| 1 - (Reciprocal + 1) Model | .12 | -76956 | -76882 | 38452 | 7 |  |  |
| Log+1 Model | .11 | 303874 | 303947 | -151959 | 7 | 380823 | .000 |
| Poisson Model | .09 | 979581 | 979645 | -489785 | 6 | 675651 | .000 |
| Negative Binomial Model | .03 | 411925 | 411999 | -205956 | 7 | 567658 | .000 |

*Notes.* Model fit was calculated using the R package lmtest (Hothorn et al., 2015)

## Hillary election loss

**Table 12S. Linear regression 1-(reciprocal+1) model:** Linear mixed model with four factors (positive language, negative language, number of followers, positive language x negative language) and number of retweets 1-(reciprocal+1) as the dependent variable.

| Fixed Effects | | | | | |
| --- | --- | --- | --- | --- | --- |
|  | Estimate | *SE* | 95% CI | *t* | *p* |
| Intercept | 0.16 | 0.00077 | 0.16 – 0.16 | 208.34 | .000 |
| Positive Language | 0.0010 | 0.00080 | -0.00072 – 0.0025 | 1.25 | .209 |
| Negative Language | 0.0060 | 0.00045 | 0.0049 – 0.0069 | 13.45 | .000 |
| Number of followers | 0.072 | 0.00059 | 0.070 – 0.073 | 121.88 | .000 |
| Positive Language × Negative Language | 0.00065 | 0.00054 | -0.00031– 0.0019 | 1.21 | .219 |
| Random Effects | | | | | |
|  | |  | Variance | *SD* |  |
| Participant (Intercept) | |  | 0.019 | 0.14 |  |
| Residual | |  | 0.046 | 0.21 |  |
| Model fit | | | | | |
| *R^2^* | |  | Marginal | Conditional | |
|  | |  | 0.07 | 0.35 | |
| Model equation: Retweets 1-(reciprocal+1) ~ Positive * Negative + centered (Followers) + (1 \| User) | | | | | |

*Notes.* Model fit was calculated using the R package MuMIn (Barton, 2018) based on the paper of Nakagawa et al. (2017).

**Table 13S. Linear regression (log+1) model:** Linear mixed model with four factors (positive language, negative language, number of followers, positive language x negative language) and number of retweets (log+1) as the dependent variable.

| Fixed Effects | | | | | |
| --- | --- | --- | --- | --- | --- |
|  | Estimate | *SE* | 95% CI | *t* | *p* |
| Intercept | 0.092 | 0.00056 | 0.091 – 0.093 | 163.32 | .000 |
| Positive Language | 0.00057 | 0.00056 | -0.00046 – 0.0017 | 1.02 | .309 |
| Negative Language | 0.0037 | 0.00031 | 0.0031 – 0.0043 | 12.05 | .000 |
| Number of followers | 0.054 | 0.00045 | 0.053 – 0.055 | 120.23 | .000 |
| Positive Language × Negative Language | 0.00015 | 0.00037 | -0.00053 – 0.00094 | 0.40 | .682 |
| Random Effects | | | | | |
|  | |  | Variance | *SD* |  |
| Participant (Intercept) | |  | 0.014 | 0.12 |  |
| Residual | |  | 0.02 | 0.14 |  |
| Model fit | | | | | |
| *R*^2^ | |  | Marginal | Conditional | |
|  | |  | 0.08 | 0.45 | |
| Model equation: Retweets (log+1) ~ Positive * Negative + centered (Followers) + (1 \| User) | | | | | |

*Notes.* Model fit was calculated using the R package MuMIn (Barton, 2018) based on the paper of Nakagawa et al. (2017).

**Table 14S. Poisson regression model:** Count mixed model with four factors (positive language, negative language, number of followers, positive language x negative language) and number of retweets as the dependent variable.

| Fixed Effects | | | | | |
| --- | --- | --- | --- | --- | --- |
|  | Estimate | *SE* | 95% CI | *t* | *p* |
| Intercept | -1.60 | 0.0073 | -1.69 – -1.55 | -219.94 | .000 |
| Positive Language | -0.11 | 0.0028 | -0.12 – -0.10 | -40.36 | .000 |
| Negative Language | -0.015 | 0.0058 | -0.020 – -0.0087 | -9.74 | .000 |
| Number of followers | 0.52 | 0.0018 | 0.50 – 0.53 | 89.43 | .000 |
| Positive Language × Negative Language | 0.020 | 0.0024 | 0.012 – 0.027 | -10.84 | .000 |
| Random Effects | | | | | |
|  | |  | Variance | SD |  |
| Participant (Intercept) | |  | 2.23 | 1.49 |  |
| Model fit | | | | | |
| *R*^2^ | |  | Marginal | Conditional | |
|  | |  | 0.05 | 0.44 | |
| Model equation: Retweets ~ Positive * Negative + centered (Followers) + (1 \| User), family = Poisson | | | | | |

*Notes.* Model fit was calculated using the R package MuMIn (Barton, 2018) based on the paper of Nakagawa et al. (2017).

**Table 15S. Negative binomial regression model:** Count mixed model with four factors (positive language, negative language, number of followers, positive language x negative language) and number of retweets as the dependent variable.

| Fixed Effects | | | | | |
| --- | --- | --- | --- | --- | --- |
|  | Estimate | *SE* | 95% CI | *t* | *p* |
| Intercept | -1.61 | 0.0089 | -1.64 --1.59 | -181.51 | .000 |
| Positive Language | 0.0050 | 0.0077 | -0.017 - 0.023 | 0.65 | .514 |
| Negative Language | 0.054 | 0.0042 | 0.051 - 0.058 | 12.68 | .000 |
| Number of followers | 0.67 | 0.0060 | 0.64 - 0.66 | 113.16 | .000 |
| Positive Language × Negative Language | 0.0035 | 0.0050 | -0.0050 - 0.017 | 0.69 | .490 |
| Random Effects | | | | | |
|  | |  | Variance | *SD* |  |
| Participant (Intercept) | |  | 1.76 | 1.32 |  |
| Model fit | | | | | |
| R^2^ | |  | Marginal | Conditional | |
|  | |  | .03 | .16 | |
| Model equation: Retweets ~ Positive * Negative + centered (Followers) + (1 \| User), family = Poisson | | | | | |

*Notes.* Model fit was calculated using the R package MuMIn (Barton, 2018) based on the paper of Nakagawa et al. (2017).

**Table 16S. Model Comparison:** Comparing log-likelihood scores of the above described models.

|  | Marginal *R*^2^ | AIC | BIC | Log-Likelihood | df | chi-squared | *p* |
| --- | --- | --- | --- | --- | --- | --- | --- |
| 1 - (Reciprocal + 1) Model | .07 | 2706 | 2781 | -1379 | 7 |  |  |
| Log+1 Model | .08 | -241976 | 303947 | 120960 | 7 | 244679 | .000 |
| Poisson Model | .05 | 1467897 | 1467961 | -733942 | 6 | 1709805 | .000 |
| Negative Binomial Model | 0.3 | 651565 | 651640 | -325775 | 7 | 816334 | .000 |

*Notes.* Model fit was calculated using the R package lmtest (Hothorn et al., 2015)]

## Same-sex ruling

**Table 17S. Linear regression 1-(reciprocal+1) model:** Linear mixed model with four factors (positive language, negative language, number of followers, positive language x negative language) and number of retweets 1-(reciprocal+1) as the dependent variable.

| Fixed Effects | | | | | |
| --- | --- | --- | --- | --- | --- |
|  | Estimate | *SE* | 95% CI | *t* | *p* |
| Intercept | 0.12 | 0.00050 | 0.12 – 0.12 | 241.28 | .000 |
| Positive Language | 0.0014 | 0.00035 | 0.00075 – 0.0020 | 4.02 | .000 |
| Negative Language | 0.0063 | 0.00051 | 0.0055 – 0.0075 | 12.43 | .000 |
| Number of followers | 0.097 | 0.00034 | 0.096 - 0.097 | 283.07 | .000 |
| Positive Language × Negative Language | 0.00057 | 0.00039 | -0.00024 - 0.0012 | 1.45 | .145 |
| Random Effects | | | | | |
|  | |  | Variance | *SD* |  |
| Participant (Intercept) | |  | 0.013 | 0.11 |  |
| Residual | |  | 0.034 | 0.18 |  |
| Model fit | | | | | |
| *R*^2^ | |  | Marginal | Conditional | |
|  | |  | .16 | .40 | |
| Model equation: Retweets 1-(reciprocal+1) ~ Positive * Negative + centered (Followers) + (1 \| User) | | | | | |

*Notes.* Model fit was calculated using the R package MuMIn (Barton, 2018) based on the paper of Nakagawa et al. (2017).

**Table 18S. Linear regression (log+1) model:** Linear mixed model with four factors (positive language, negative language, number of followers, positive language x negative language) and number of retweets (log+1) as the dependent variable.

| Fixed Effects | | | | | |
| --- | --- | --- | --- | --- | --- |
|  | Estimate | *SE* | 95% CI | *t* | *p* |
| Intercept | 0.21 | 0.0010 | 0.21 – 0.21 | 201.85 | .000 |
| Positive Language | 0.0035 | 0.00072 | 0.0023 - 0.0050 | 4.93 | .309 |
| Negative Language | 0.0097 | 0.0010 | 0.0078 - 0.011 | 9.31 | .000 |
| Number of followers | 0.22 | 0.00072 | 0.22 - 0.22 | 307.05 | .000 |
| Positive Language × Negative Language | 0.0010 | 0.00079 | -0.00046 - 0.0022 | 1.36 | .172 |
| Random Effects | | | | | |
|  | |  | Variance | *SD* |  |
| Participant (Intercept) | |  | 0.0085 | 0.092 |  |
| Residual | |  | 0.014 | 0.12 |  |
| Model fit | | | | | |
| *R*^2^ | |  | Marginal | Conditional | |
|  | |  | .19 | .49 | |
| Model equation: Retweets (log+1) ~ Positive * Negative + centered (Followers) + (1 \| User) | | | | | |

*Notes.* Model fit was calculated using the R package MuMIn (Barton, 2018) based on the paper of Nakagawa et al. (2017).

**Table 19S. Poisson regression model:** Count mixed model with four factors (positive language, negative language, number of followers, positive language x negative language) and number of retweets as the dependent variable.

| Fixed Effects | | | | | |
| --- | --- | --- | --- | --- | --- |
|  | Estimate | SE | 95% CI | *t* | *p* |
| Intercept | -2.17 | 0.0062 | -2.17 – -2.16 | -349.68 | .000 |
| Positive Language | -0.065 | 0.0016 | 0.070 - -0.060 | -38.97 | .000 |
| Negative Language | -0.15 | 0.0030 | -0.15 - -0.14 | -50.50 | .000 |
| Number of followers | 1.09 | 0.0041 | 1.08 - 1.10 | 262.14 | .000 |
| Positive Language × Negative Language | 0.17 | 0.0023 | 0.17 - 0.17 | 73.70 | .000 |
| Random Effects | | | | | |
|  | |  | Variance | *SD* |  |
| Participant (Intercept) | |  | 1.95 | 1.40 |  |
| Model fit | | | | | |
| *R*^2^ | |  | Marginal | Conditional | |
|  | |  | .14 | .39 | |
| Model equation: Retweets ~ Positive * Negative + centered (Followers) + (1 \| User), family = Poisson | | | | | |

*Notes.* Model fit was calculated using the R package MuMIn (Barton, 2018) based on the paper of Nakagawa et al. (2017).

**Table 20S. Negative binomial regression model:** Count mixed model with four factors (positive language, negative language, number of followers, positive language x negative language) and number of retweets as the dependent variable.

| Fixed Effects | | | | | |
| --- | --- | --- | --- | --- | --- |
|  | Estimate | *SE* | 95% CI | *t* | *p* |
| Intercept | -2.16 | 0.0077 | -2.17 - -2.16 | -280.39 | .000 |
| Positive Language | 0.0050 | 0.0042 | -0.0036 - 0.012 | 1.19 | .232 |
| Negative Language | 0.054 | 0.0058 | 0.048 - 0.058 | 9.24 | .000 |
| Number of followers | 1.081 | 0.0041 | 1.054 - 1.076 | 259.49 | .000 |
| Positive Language × Negative Language | 0.012 | 0.0044 | 0.0091 - 0.017 | 2.75 | .006 |
| Random Effects | | | | | |
|  | |  | Variance | *SD* |  |
| Participant (Intercept) | |  | 1.60 | 1.26 |  |
| Model fit | | | | | |
| *R*^2^ | |  | Marginal | Conditional | |
|  | |  | .027 | .06 | |
| Model equation: Retweets ~ Positive * Negative + centered (Followers) + (1 \| User), family = Poisson | | | | | |

*Notes.* Model fit was calculated using the R package MuMIn (Barton, 2018) based on the paper of Nakagawa et al. (2017).

**Table 21S. Model Comparison:** Comparing log-likelihood scores of the above described models.

|  | Marginal *R*^2^ | AIC | BIC | Log-Likelihood | df | chi-squared | *p* |
| --- | --- | --- | --- | --- | --- | --- | --- |
| 1 - (Reciprocal + 1) Model | .16 | -139750 | -139671 | 69882 | 7 |  |  |
| Log+1 Model | .19 | -572045 | -571966 | 286029 | 7 | 432291 | .000 |
| Poisson Model | .15 | 1430343 | 1430411 | -715166 | 6 | 2002316 | .000 |
| Negative Binomial Model | .027 | 805288 | 805367 | -402637 | 7 | 625057 | .000 |

*Notes.* Model fit was calculated using the R package lmtest (Hothorn et al., 2015)

## Ferguson unrest

**Table 22S. Linear regression 1-(reciprocal+1) model:** Linear mixed model with four factors (positive language, negative language, number of followers, positive language x negative language) and number of retweets 1-(reciprocal+1) as the dependent variable.

| Fixed Effects | | | | | |
| --- | --- | --- | --- | --- | --- |
|  | Estimate | *SE* | 95% CI | *t* | *p* |
| Intercept | 0.18 | 0.00064 | 0.17 – 0.18 | 279.78 | .000 |
| Positive Language | -0.0011 | 0.00072 | -0.0026 - 0.00019 | -1.53 | .124 |
| Negative Language | 0.0079 | 0.00036 | 0.0071 - 0.0087 | 21.56 | .000 |
| Number of followers | 0.099 | 0.00051 | 0.097 - 0.10 | 192.47 | .000 |
| Positive Language × Negative Language | -0.00054 | 0.00051 | -0.0016 - 0.00026 | -1.05 | .291 |
| Random Effects | | | | | |
|  | |  | Variance | *SD* |  |
| Participant (Intercept) | |  | 0.013 | 0.12 |  |
| Residual | |  | 0.054 | 0.23 |  |
| Model fit | | | | | |
| *R*^2^ | |  | Marginal | Conditional | |
|  | |  | .13 | .29 | |
| Model equation: Retweets 1-(reciprocal+1) ~ Positive * Negative + centered (Followers) + (1 \| User) | | | | | |

*Notes.* Model fit was calculated using the R package MuMIn (Barton, 2018) based on the paper of Nakagawa et al. (2017).

**Table 23S. Linear regression (log+1) model:** Linear mixed model with four factors (positive language, negative language, number of followers, positive language x negative language) and number of retweets (log+1) as the dependent variable.

| Fixed Effects | | | | | |
| --- | --- | --- | --- | --- | --- |
|  | Estimate | *SE* | 95% CI | *t* | *p* |
| **Intercept** | 0.30 | 0.0013 | 0.30 – 0.30 | 221.32 | .000 |
| **Positive Language** | -0.0046 | 0.0015 | -0.0083 - -0.0021 | -3.09 | .002 |
| **Negative Language** | 0.016 | 0.00077 | 0.015 - 0.018 | 21.89 | .000 |
| **Number of followers** | 0.21 | 0.0011 | 0.21 - 0.21 | 191.53 | .000 |
| **Positive Language x Negative Language** | -0.000032 | 0.0010 | -0.0024 - 0.0019 | -0.03 | .976 |
| Random Effects | | | | | |
|  | |  | Variance | *SD* |  |
| Participant (Intercept) | |  | 0.07 | 0.26 |  |
| Residual | |  | 0.23 | 0.48 |  |
| Model fit | | | | | |
| *R*^2^ | |  | Marginal | Conditional | |
|  | |  | .13 | .33 | |
| Model equation: Retweets (log+1) ~ Positive * Negative + centered (Followers) + (1 \| User) | | | | | |

*Notes.* Model fit was calculated using the R package MuMIn (Barton, 2018) based on the paper of Nakagawa et al. (2017).

**Table 24S. Poisson regression model:** Count mixed model with four factors (positive language, negative language, number of followers, positive language x negative language) and number of retweets as the dependent variable.

| Fixed Effects | | | | | |
| --- | --- | --- | --- | --- | --- |
|  | Estimate | *SE* | 95% CI | *t* | *p* |
| Intercept | -1.39 | 0.0055 | -1.39 – -1.38 | - 253.52 | .000 |
| Positive Language | -0.073 | 0.0025 | -0.083 - -0.061 | -28.68 | .000 |
| Negative Language | 0.063 | 0.0011 | 0.060 - 0.067 | 52.95 | .000 |
| Number of followers | 0.73 | 0.0040 | 0.72 - 0.74 | 183.09 | .000 |
| Positive Language × Negative Language | 0.036 | 0.0017 | 0.031 - 0.040 | 20.69 | .000 |
| Random Effects | | | | | |
|  | |  | Variance | *SD* |  |
| Participant (Intercept) | |  | 1.39 | 1.18 |  |
| Model fit | | | | | |
| *R*^2^ | |  | Marginal | Conditional | |
|  | |  | .09 | .33 | |
| Model equation: Retweets ~ Positive * Negative + centered (Followers) + (1 \| User), family = Poisson | | | | | |

*Notes.* Model fit was calculated using the R package MuMIn (Barton, 2018) based on the paper of Nakagawa et al. (2017).

**Table 25S. Negative binomial regression model:** Count mixed model with four factors (positive language, negative language, number of followers, positive language x negative language) and number of retweets as the dependent variable.

| 1. Fixed Effects | | | | | |
| --- | --- | --- | --- | --- | --- |
|  | Estimate | *SE* | 95% CI | *t* | *p* |
| Intercept | -1.27 | 0.0064 | -1.28 - -1.26 | -197.14 | .000 |
| Positive Language | -0.014 | 0.0059 | -0.020 - -0.0053 | -2.42 | .015 |
| Negative Language | 0.065 | 0.0029 | 0.055 - 0.070 | 21.84 | .000 |
| Number of followers | 0.85 | 0.0048 | 0.82 - 0.84 | 177.44 | .000 |
| Positive Language × Negative Language | -0.0032 | 0.0042 | -0.011 - -0.00075 | -0.76 | .445 |
| Random Effects | | | | | |
|  | |  | Variance | *SD* |  |
| Participant (Intercept) | |  | 0.99 | 0.99 |  |
| Model fit | | | | | |
| *R*^2^ | |  | Marginal | Conditional | |
|  | |  | .04 | .09 | |
| Model equation: Retweets ~ Positive * Negative + centered (Followers) + (1 \| User), family = Poisson | | | | | |

*Notes.* Model fit was calculated using the R package MuMIn (Barton, 2018) based on the paper of Nakagawa et al. (2017).

**Table 26S. Model Comparison:** Comparing log-likelihood scores of the above described models.

|  | Marginal *R^2^* | AIC | BIC | Log-Likelihood | df | chi-squared | *p* |
| --- | --- | --- | --- | --- | --- | --- | --- |
| 1-(Reciprocal + 1) Model | .13 | 40115 | 40193 | -20084 | 7 |  |  |
| Log+1 Model | .13 | 862078 | 862157 | -431062 | 7 | 821956 | .000 |
| Poisson Model | .09 | 2567482 | 2567550 | -1283735 | 6 | 1705346 | .000 |
| Negative Binomial Model | .04 | 1180476 | 1180554 | -590231 | 7 | 1387009 | .000 |

*Notes.* Model fit was calculated using the R package lmtest (Hothorn et al., 2015)

# **Emotional language intensity predicting retweets: General Additive Models**

To test if linear parametric models are the appropriate choice we ran general additive mixed model via mgcv package in R (Wood, 2015). As in the linear mixed model, we predicted the 1-(reciprocal+1) transformed retweet count using positive and negative SentiStrength scores. The number of knots for both positive and negative scores were 4. The results supported the initial claim that negativity spreads further than positivity in all situations.

## Trump election win

**Table 27S. GAM 1-(reciprocal+1) model:** General additive model with two smooth factors (positive language, negative language) using 4 knots and one parametric factor (number of followers) predicting number of retweets 1-(reciprocal+1) as the dependent variable.

| Non-Parametric Terms | | | | | |
| --- | --- | --- | --- | --- | --- |
|  | Estimate | *SE* | | *t* | *p* |
| Intercept | -0.098 | 0.0018 | | -53.38 | .000 |
| Number of followers | 0.039 | 0.00031 | | 124.61 | .000 |
| Smooth-Terms | | | | | |
|  | edf | Ref.df | | *F* | *p* |
| Positive Language (4 knots) | 1.00 | 1.00 | | 1.25 | .884 |
| Negative Language (4 knots) | 2.443 | 2.443 | | 6.86 | .000 |
| Random Effects | | | | | |
|  | |  | Variance | *SD* |  |
| Participant (Intercept) | |  | 0.017 | 0.13 |  |
| Residual | |  | 0.034 | 0.18 |  |
| Model fit | | | | | |
| *R*^2^ | |  |  | Adjusted | |
|  | |  |  | .14 | |
| Model equation: Retweets 1-(reciprocal+1) ~ (Positive, knots = 4) + (Negative, knots = 4) + centered (Followers) + (1 \| User) | | | | | |

*Notes.* General additive mixed model was computed via mgcv package in R (Wood, 2015).


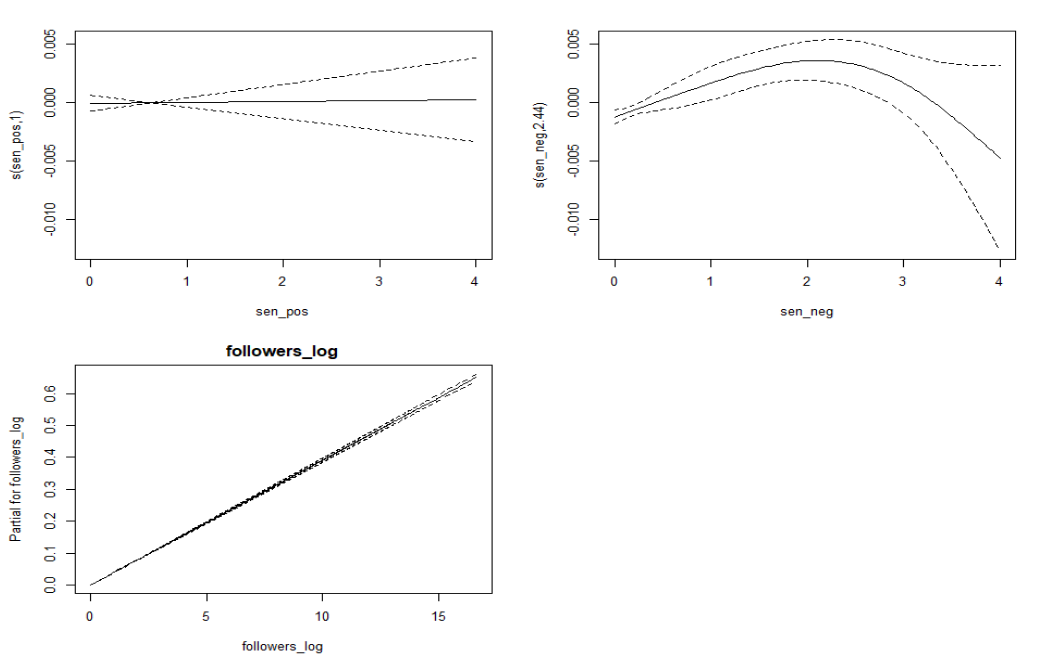


**Figure 7S.** Results of emotional language intensity (negative and positive) predicting number of retweets (1-(reciprocal+1) transformed) expressed in the election win dataset. We found that an increase in negative language intensity is associated with an increase in the number of retweets, while an increase in positive language was not correlated with number of retweets. It is noteworthy, that for very intense negative scores the prediction is imprecise indicated by the large confidence intervals.

## Hillary election loss

**Table 28S. GAM 1-(reciprocal+1) model:** General additive model with two smooth factors (positive language, negative language) using 4 knots and one parametric factor (number of followers) predicting number of retweets 1-(reciprocal+1) as the dependent variable.

| Non-Parametric Terms | | | | | |
| --- | --- | --- | --- | --- | --- |
|  | Estimate | *SE* | | *t* | *p* |
| Intercept | -0.019 | 0.0016 | | -11.96 | .000 |
| Number of followers | 0.033 | 0.00027 | | 121.84 | .000 |
| Smooth-Terms | | | | | |
|  | edf | Ref.df | | *F* | *p* |
| Positive Language (4 knots) | 1.00 | 1.00 | | 8.92 | .002 |
| Negative Language (4 knots) | 2.877 | 2.877 | | 137.19 | .000 |
| Random Effects | | | | | |
|  | |  | Variance | *SD* |  |
| Participant (Intercept) | |  | 0.046 | 0.21 |  |
| Residual | |  | 0.019 | 0.13 |  |
| Model fit | | | | | |
| *R*^2^ | |  |  | Adjusted | |
|  | |  |  | .09 | |
| Model equation: Retweets 1-(reciprocal+1) ~ (Positive, knots = 4) + (Negative, knots = 4) + centered (Followers) + (1 \| User) | | | | | |

*Notes.* General additive mixed model was computed via mgcv package in R (Wood, 2015).


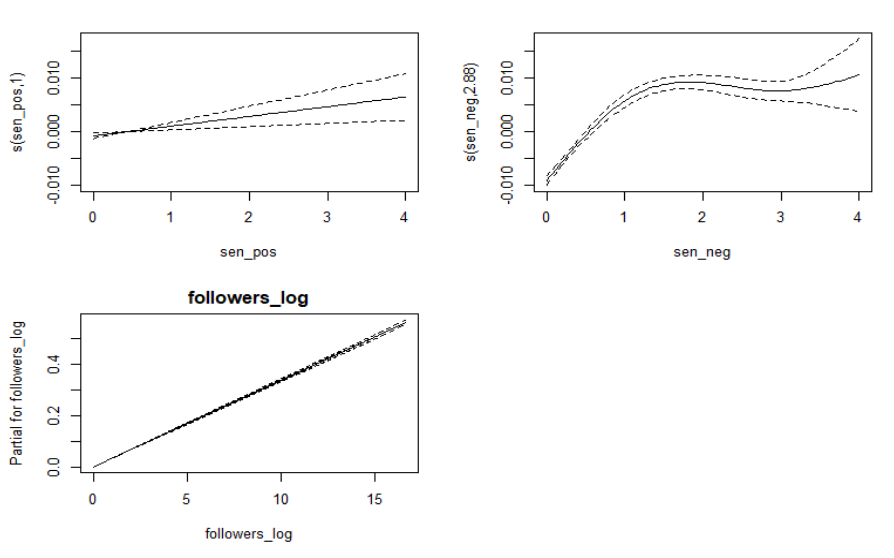


**Figure 8S.** Results of emotional language intensity (negative and positive) predicting number of retweets (1-(reciprocal+1) transformed) expressed in the election win dataset. We found that an increase in negative language intensity is associated with an increase in the number of retweets. An increase in positive language was also correlated with number of retweets.

## Same-sex marriage

**Table 29S. GAM 1-(reciprocal+1) model:** General additive model with two smooth factors (positive language, negative language) using 4 knots and one parametric factor (number of followers) predicting number of retweets 1-(reciprocal+1) as the dependent variable.

| Non-Parametric Terms | | | | | |
| --- | --- | --- | --- | --- | --- |
|  | Estimate | *SE* | | *t* | *p* |
| Intercept | -0.22 | 0.0012 | | -181.0 | .000 |
| Number of followers | 0.055 | 0.00019 | | 283.2 | .000 |
| Smooth-Terms | | | | | |
|  | edf | Ref.df | | *F* | *p* |
| Positive Language (4 knots) | 2.99 | 2.99 | | 134.0 | .002 |
| Negative Language (4 knots) | 2.75 | 2.75 | | 154.7 | .000 |
| Random Effects | | | | | |
|  | |  | Variance | *SD* |  |
| Participant (Intercept) | |  | 0.013 | 0.11 |  |
| Residual | |  | 0.034 | 0.18 |  |
| Model fit | | | | | |
| *R*^2^ | |  |  | Adjusted | |
|  | |  |  | .18 | |
| Model equation: Retweets 1-(reciprocal+1) ~ (Positive, knots = 4) + (Negative, knots = 4) + centered (Followers) + (1 \| User) | | | | | |

*Notes.* General additive mixed model was computed via mgcv package in R (Wood, 2015).


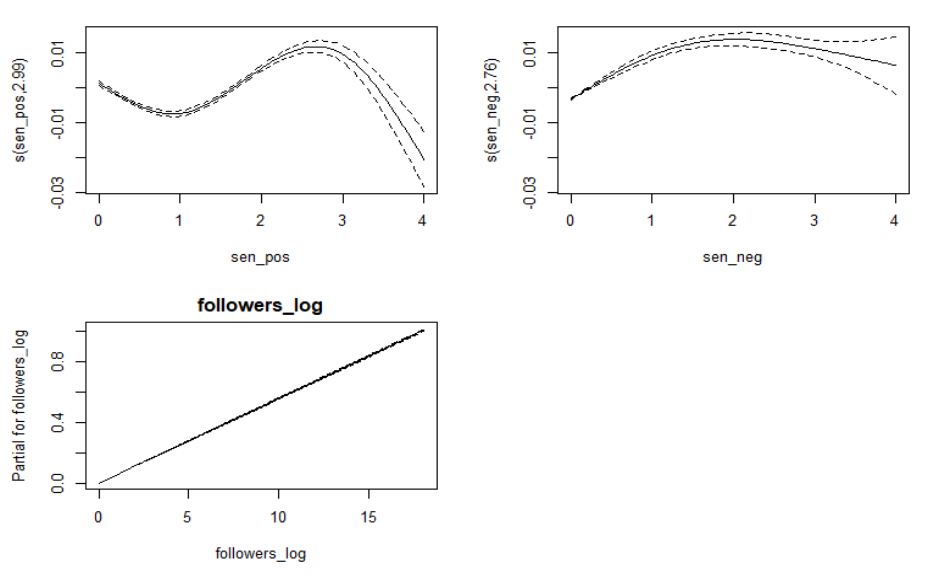


**Figure 9S.** Results of emotional language intensity (negative and positive) predicting number of retweets (1-(reciprocal+1) transformed) expressed in the election win dataset. We found that an increase in negative language intensity is associated with an increase in the number of retweets. An increase in positive language was also correlated with number of retweets. However, it is important to note that the positive language score is not strictly linear suggesting that for very intense positive emotions there was a negative association with number of retweets.

## Ferguson unrest

**Table 30S. GAM 1-(reciprocal+1) model:** General additive model with two smooth factors (positive language, negative language) using 4 knots and one parametric factor (number of followers) predicting number of retweets 1-(reciprocal+1) as the dependent variable.

| 1. Non-Parametric Terms | | | | | |
| --- | --- | --- | --- | --- | --- |
|  | Estimate | *SE* | | *t* | *p* |
| **Intercept** | -0.14 | 0.0017 | | -86.33 | .000 |
| **Number of followers** | 0.053 | 0.00027 | | 192.41 | .000 |
| 1. Smooth-Terms | | | | | |
|  | edf | Ref.df | | *F* | *p* |
| **Positive Language (4 knots)** | 1.85 | 1.85 | | 3.66 | .016 |
| **Negative Language (4 knots)** | 2.93 | 2.93 | | 277.46 | .000 |
| Random Effects | | | | | |
|  | |  | Variance | *SD* |  |
| Participant (Intercept) | |  | 0.013 | 0.11 |  |
| Residual | |  | 0.054 | 0.23 |  |
| Model fit | | | | | |
| *R*^2^ | |  |  | Adjusted | |
|  | |  |  | .16 | |
| Model equation: Retweets 1-(reciprocal+1) ~ (Positive, knots = 4) + (Negative, knots = 4) + centered (Followers) + (1 \| User) | | | | | |

*Notes.* General additive mixed model was computed via mgcv package in R (Wood, 2015).


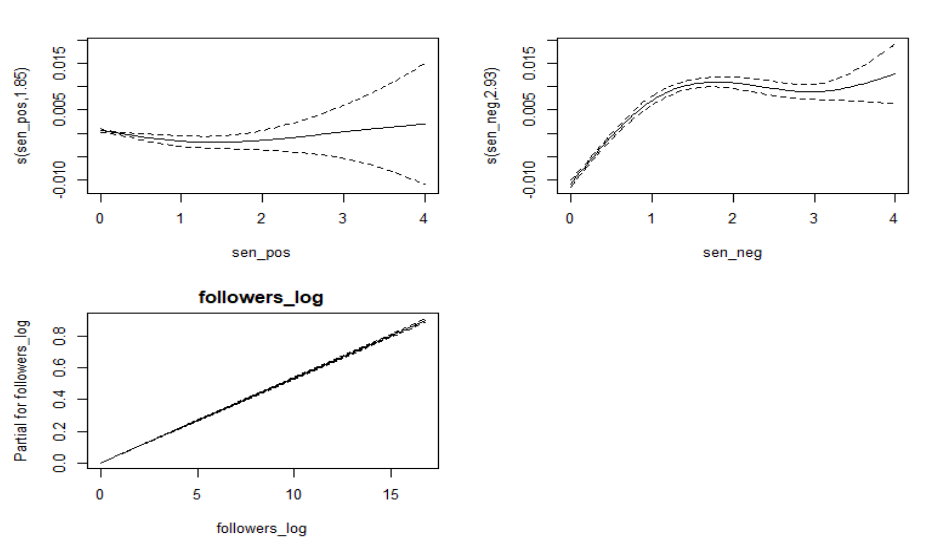


**Figure 10S.** Results of emotional language intensity (negative and positive) predicting number of retweets (1-(reciprocal+1) transformed) expressed in the election win dataset. We found that an increase in negative language intensity is associated with an increase in the number of retweets. An increase in positive language was not correlated with number of retweets. It is noteworthy, that for very intense negative scores the prediction is imprecise indicated by the large confidence intervals.

# **Emotional language intensity predicting retweets: Sentiment Tool Comparison**

To test whether SentiStrenght is the best choice of sentiment analysis tool for the political situations we chose, we compared SentiStrength against VADER (Gilbert & Hutto, 2014). We first correlated both scores.

- 1. Election Win (Positive Emotion *r* = 52, Negative Emotion  *r* = .55),
  2. Election Loss (Positive Emotion *r* = .55, Negative Emotion  *r* =.62),
  3. Same Sex Ruling (Positive Emotion *r* = .60, Negative Emotion  *r* = .60),
  4. Ferguson (Positive Emotion *r* = .53, Negative Emotion  *r* = .63)

After that we ran the identical analysis as described above using the following formula:

Lmer (reciprocal(retweet+1) ~

positive_score + negative_vader + positive_vader *negative_vader + (user_id),

family = “gaussian”)

Finally we compared the model fits using the log-likelihood test method from the lmtest package (Bates, Sarkar, Bates, & Matrix, 2007).

## Trump election win

**Table 31S. Linear regression 1-(reciprocal+1) model:** Linear mixed model with four factors (positive language, negative language, number of followers, positive language x negative language) and number of retweets 1-(reciprocal+1) as the dependent variable.

| Fixed Effects | | | | | |
| --- | --- | --- | --- | --- | --- |
|  | Estimate | *SE* | 95% CI | *t* | *p* |
| **Intercept** | 0.16 | 0.00080 | 0.13 - 0.13 | 165.31 | .000 |
| **Positive Language Vader** | -0.0074 | 0.0028 | -0.013 - -0.0011 | -2.56 | .010 |
| **Negative Language Vader** | 0.0023 | 0.0041 | -0.0055 - 0.010 | 0.57 | .565 |
| **Number of followers** | 0.080 | 0.00064 | 0.079 - 0.081 | 124.49 | .000 |
| **Positive Language V. x Negative Language V.** | 0.038 | 0.033 | -0.0091 - 0.099 | 1.15 | .249 |
| Random Effects | | | | | |
|  | |  | Variance | *SD* |  |
| Participant (Intercept) | |  | 0.019 | 0.13 |  |
| Residual | |  | 0.046 | 0.21 |  |
| Model fit | | | | | |
| *R*^2^ | |  | Marginal | Conditional | |
|  | |  | .11 | .41 | |
| Model equation: Retweets 1-(reciprocal+1) ~ Positive V. * Negative V. + centered (Followers) + (1 \| User) | | | | | |

*Notes.* Model fit was calculated using the R package MuMIn (Barton, 2018) based on the paper of Nakagawa et al. (2017).

**Table 32S. Model Comparison:** Comparing log-likelihood scores of SentiStrength vs. VADER models.

|  | Marginal *R*^2^ | AIC | BIC | Log-Likelihood | df | chi-squared | *p* |
| --- | --- | --- | --- | --- | --- | --- | --- |
| SentiStrength | .12 | -76956 | -76882 | 38452 | 7 |  |  |
| VADER | .11 | -76954 | -76880 | 38459 | 7 | 13.097 | .000 |

*Notes.* Model fit was calculated using the R package lmtest (Hothorn et al., 2015)

## Hillary election loss

**Table 33S. Linear regression 1-(reciprocal+1) model:** Linear mixed model with four factors (positive language, negative language, number of followers, positive language x negative language) and number of retweets 1-(reciprocal+1) as the dependent variable.

| Fixed Effects | | | | | |
| --- | --- | --- | --- | --- | --- |
|  | Estimate | *SE* | 95% CI | *t* | *p* |
| Intercept | 0.16 | 0.00080 | 0.16 - 0.16 | 204.15 | .000 |
| Positive Language Vader | -0.0053 | 0.0028 | -0.012 - 0.0017 | -1.40 | .160 |
| Negative Language Vader | 0.016 | 0.0041 | 0.0091 - 0.023 | 4.94 | .000 |
| Number of followers | 0.072 | 0.00064 | 0.070 - 0.073 | 121.68 | .000 |
| Positive Language V. × Negative Language V. | 0.24 | 0.028 | 0.18 - 0.29 | 8.68 | .000 |
| Random Effects | | | | | |
|  | |  | Variance | SD |  |
| Participant (Intercept) | |  | 0.019 | 0.14 |  |
| Residual | |  | 0.047 | 0.22 |  |
| Model fit | | | | | |
| *R*^2^ | |  | Marginal | Conditional | |
|  | |  | .07 | .34 | |
| Model equation: Retweets 1-(reciprocal+1) ~ Positive V. * Negative V. + centered (Followers) + (1 \| User) | | | | | |

*Notes.* Model fit was calculated using the R package MuMIn (Barton, 2018) based on the paper of Nakagawa et al. (2017).

**Table 34S. Model Comparison:** Comparing log-likelihood scores of SentiStrength vs. VADER models.

|  | Marginal *R*^2^ | AIC | BIC | Log-Likelihood | df | chi-squared | *p* |
| --- | --- | --- | --- | --- | --- | --- | --- |
| SentiStrength | .07 | 2706 | 2781.3 | -1379.1 | 7 |  |  |
| VADER | .07 | 2706 | 2884.7 | -1423.2 | 7 | 88.12 | .000 |

*Notes.* Model fit was calculated using the R package lmtest (Hothorn et al., 2015)

**Summary and Evaluation of Sentiment Tools for these Datasets.**

The results for this analysis using VADER are identical in direction for the negative situations. The results for the election win were in the same direction to those reported in the manuscript but were not significant. The model fit suggested that the SentiStrength model were better in terms of their AIC and loglikelihood. As for the same sex marriage data, results did suggest that decreased negativity predicted retweets, but that positivity was also a significant negative predictor. Here again, our SentiStrength model was a better fit. One reason for VADER´s lower fit could be the way that positive and negative scores are computed. Vader has a positive, negative and a neutral score that are dependent on each other. These scores have to add up to 1, meaning that if a text contains many positive and negative words the score has to be divided between the three categories. We argue that especially in contexts that can feature both positive and negative emotions at the same time, rating positivity and negativity separately, as done by SentiStrength is crucial. The above analysis can again be found in the OSF data analysis (https://osf.io/xqevy/files) and Supplementary Materials.

## Same-sex ruling

**Table 35S. Linear regression 1-(reciprocal+1) model:** Linear mixed model with four factors (positive language, negative language, number of followers, positive language x negative language) and number of retweets 1-(reciprocal+1) as the dependent variable.

| Fixed Effects | | | | | |
| --- | --- | --- | --- | --- | --- |
|  | Estimate | *SE* | 95% CI | *t* | *p* |
| Intercept | 0.13 | 0.00052 | 0.13 - 0.14 | 261.04 | .000 |
| Positive Language Vader | -0.038 | 0.0014 | -0.04 - -0.035 | -27.46 | .160 |
| Negative Language Vader | -0.0078 | 0.0035 | -0.018 - 0.00045 | -2.19 | .028 |
| Number of followers | 0.096 | 0.00034 | 0.096 - 0.097 | 282.64 | .000 |
| Positive Language V. × Negative Language V. | 0.21 | 0.021 | 0.17- 0.25 | 9.98 | .000 |
| Random Effects | | | | | |
|  | |  | Variance | *SD* |  |
| Participant (Intercept) | |  | 0.014 | 0.11 |  |
| Residual | |  | 0.034 | 0.19 |  |
| Model fit | | | | | |
| *R*^2^ | |  | Marginal | Conditional | |
|  | |  | .16 | .40 | |
| Model equation: Retweets 1-(reciprocal+1) ~ Positive V. * Negative V. + centered (Followers) + (1 \| User) | | | | | |

*Notes.* Model fit was calculated using the R package MuMIn (Barton, 2018) based on the paper of Nakagawa et al. (2017).

**Table 36S. Model Comparison:** Comparing log-likelihood scores of SentiStrength vs. VADER models.

|  | Marginal *R*^2^ | AIC | BIC | Log-Likelihood | df | chi-squared | *p* |
| --- | --- | --- | --- | --- | --- | --- | --- |
| SentiStrength |  | -139750 | -139671 | 69847 | 7 |  |  |
| VADER | .16 | 140253 | -140174 | 70106 | 7 | 88.12 | .000 |

*Notes.* *Notes.* Model fit was calculated using the R package lmtest (Hothorn et al., 2015)

## Ferguson unrest

**Table 37S. Linear regression 1-(reciprocal+1) model:** Linear mixed model with four factors (positive language, negative language, number of followers, positive language × negative language) and number of retweets 1-(reciprocal+1) as the dependent variable.

| 1. Fixed Effects | | | | | |
| --- | --- | --- | --- | --- | --- |
|  | Estimate | *SE* | 95% CI | *t* | *p* |
| Intercept | 0.18 | 0.00067 | 0.18 - 0.19 | 276.92 | .000 |
| Positive Language Vader | -0.025 | 0.0034 | -0.032 - -0.018 | -7.45 | .160 |
| Negative Language Vader | 0.010 | 0.0029 | -0.0048 - 0.016 | 3.61 | .000 |
| Number of followers | 0.099 | 0.00051 | 0.098 - 0.10 | 192.11 | .000 |
| Positive Language V. × Negative Language V. | 0.26 | 0.028 | 0.20 - 0.32 | 9.19 | .000 |
| Random Effects | | | | | |
|  | |  | Variance | SD |  |
| Participant (Intercept) | |  | 0.013 | 0.12 |  |
| Residual | |  | 0.054 | 0.23 |  |
| Model fit | | | | | |
| *R*^2^ | |  | Marginal | Conditional | |
|  | |  | .11 | .15 | |
| Model equation: Retweets 1-(reciprocal+1) ~ Positive V. * Negative V. + centered (Followers) + (1 \| User) | | | | | |

*Notes.* Model fit was calculated using the R package MuMIn (Barton, 2018) based on the paper of Nakagawa et al. (2017).

**Table 38S. Model Comparison:** Comparing log-likelihood scores of SentiStrength vs. VADER models.

|  | Marginal *R*^2^ | AIC | BIC | Log-Likelihood | df | chi-squared | *p* |
| --- | --- | --- | --- | --- | --- | --- | --- |
| SentiStrength |  | 40115 | 40193 | -20084 | 7 |  |  |
| VADER | .16 | 40513 | 40591 | -20275 | 7 | 382.17 | .000 |

*Notes.* *Notes.* Model fit was calculated using the R package lmtest (Hothorn et al., 2015)

# **Analysis of most viral negative tweets in positive contexts**

We added the 10 most viral negative tweets to the supplementary material. We first categorized the tweets similarly to the topic model procedure, meaning the negative score has to be higher than the positive score. After that, we sorted them by number of retweets. The 10 most viral tweets in both situations were:

## Trump election win

**Table 39S. Most viral tweets:** Tweets with a negative sentiment score sorted by number of retweets.

| No. | Tweet content | Retweets |
| --- | --- | --- |
| 1. | They laughed at us when we said #realDonaldTrump would win. This morning they’re too devastated to get out of bed | 5759 |
| 2. | President elect Trump did what Obama never would he called families of slain officers to offer condolences | 5606 |
| 3. | If you think they hate me now wait until I keep all my campaign promises #MAGA | 4906 |
| 4. | We The People are awake amp we will expose amp vote out Establishment corruption #DrainTheSwamp #MAGA 2018 | 4315 |
| 5. | Soros Scrambling To Cover Up Evil Atheist Video From 1998 SEE IT BEFORE ITS DELETED #MAGA #AmericaFirst #Dobbs | 4210 |
| 6. | America Is BURNING Property Is Being DESTROYED People are Being ATTACKED Barrys Silence Equals Consent #MAGA | 4155 |
| 7. | Hillary conceded the election so that means that shes lied about absolutely everything this year #MAGA #AmericaFirst #realDonaldTrump | 3201 |
| 8. | DonaldTrump didnt win today Hate won Fear won Racism won Sexism won Homophobia won Prejudice won | 2536 |
| 9. | This scare me Trump #TheSimpsons #PresidentTrump #TrumpPresident #TrumpsFirstOrder LoPrimeroQueHaráTrump | 2709 |
| 10. | A protest is an objection to a grievance If its just a bunch of sore losers. it’s called a tantrum #MAGA | 2709 |

## Same-sex ruling

**Table 40S. Most viral tweets:** Tweets with a negative sentiment score sorted by number of retweets.

| No. | Tweet content | Retweets |
| --- | --- | --- |
| 1. | today is honestly such a big day in history and we shouldnt forget how far we have come and all ppl have done to fight for equality #LoveWins | 24017 |
| 2. | i cried #lovewins #happybirthdaytome #proudtobeanamerican af | 17602 |
| 3. | before you decide to be an asshole just remember that there are people that have waited 50 years for today #LoveWins | 2536 |
| 4. | On this day #LoveWins Gallavich Shameless Showtime | 2457 |
| 5. | This church has a message for hateful members of their church #LoveWins | 1433 |
| 6. | While today’s decision is historic. We can’t stop working until weve outlawed discrimination in every corner of our country #LoveWins HRC | 1387 |
| 7. | Angry Americans saying they will flee to Canada hate to break it to you but #LoveWins there too | 1342 |
| 8. | The biggest world news today were the attacks in Tunisia Kuwait and France The biggest news on twitter was #LoveWins of America | 1193 |
| 9. | Fuck yeah #LoveWins | 1035 |
| 10. | This ruling is a victory for gay and lesbian couples who have fought so long for their basic civil rights POTUS #LoveWins | 879 |

We believe that this content mostly is congruent with the claim we made in the manuscript that negativity was mostly directed at the opposing group or the challenging path that the group has gone through to get to the celebrated moment. While there is an aspect of celebration in many of these tweets, there are also an abundance of negative emotions.

# **Removing emotional ambiguous words from same-sex dataset**

Similar to the initial topic modelling analysis, it seems that some of the negative tweets response to the same sex marriage are still expressing positive effect. One example is tweet No.2 table 39S. (“I **cried** #lovewins #happybirthdaytome #proudtobeanamerican af”). Our second attempt to account for this limitation was to redo our analysis after removing all of the tweets who had the terms “cry”, “sob”, and “tear”. After removing these tweets from the dataset we conducted the same analysis. Results shows similar effects to our main analysis, suggesting that the effect of these tweets on the over conclusion is marginal. We again used the same model as described above.

## Same-sex ruling

**Table 41S. Linear regression 1-(reciprocal+1) model:** Linear mixed model with four factors (positive language, negative language, number of followers, positive language x negative language) and number of retweets 1-(reciprocal+1) as the dependent variable.

| Fixed Effects | | | | | |
| --- | --- | --- | --- | --- | --- |
|  | Estimate | *SE* | 95% CI | *t* | *p* |
| **Intercept** | 0.12 | 0.0016 | 0.13 - 0.14 | 72.56 | .000 |
| **Positive Language Vader** | 0.00019 | 0.00076 | -0.04 - -0.035 | 0.26 | .794 |
| **Negative Language Vader** | 0.0086 | 0.0011 | -0.018 - 0.00045 | 7.89 | .000 |
| **Number of followers** | 0.094 | 0.00036 | 0.096 - 0.097 | 259.85 | .000 |
| **Positive Language V. x Negative Language V.** | 0.00099 | 0.00051 | 0.17- 0.25 | 1.93 | .052 |
| Random Effects | | | | | |
|  | |  | Variance | *SD* |  |
| Participant (Intercept) | |  | 0.020 | 0.14 |  |
| Residual | |  | 0.042 | 0.20 |  |
| Model fit | | | | | |
| *R*^2^ | |  | Marginal | Conditional | |
|  | |  | .13 | .41 | |
| Model equation: Retweets 1-(reciprocal+1) ~ Positive V. * Negative V. + centered (Followers) + (1 \| User) | | | | | |

*Notes.* Model fit was calculated using the R package MuMIn (Barton, 2018) based on the paper of Nakagawa et al. (2017).

# **Reference**

Bates, D., Sarkar, D., Bates, M. D., & Matrix, L. (2007). The lme4 package. *2*(1), 74.

Gilbert, C., & Hutto, E. (2014). *Vader: A parsimonious rule-based model for sentiment analysis of social media text.* Paper presented at the Eighth International Conference on Weblogs and Social Media (ICWSM-14). Available at (20/04/16) <http://comp>. social. gatech. edu/papers/icwsm14. vader. hutto. pdf.

Hothorn, T., Zeileis, A., Farebrother, R. W., Cummins, C., Millo, G., Mitchell, D., & Zeileis, M. A. (2015). Package ‘lmtest’. *Testing linear regression models r-project* [*https://cran*](https://cran)*. r-project. org/web/packages/lmtest/lmtest. pdf. , 6*.

Wood, S. (2015). Package ‘mgcv’. *R package version, 1*, 29.
